# Helically chiral multiresonant thermally activated delayed fluorescent emitters and their use in hyperfluorescent organic light-emitting diodes†

**DOI:** 10.1039/d4sc03478c

**Published:** 2024-09-18

**Authors:** Jingxiang Wang, Dongyang Chen, Juan Manuel Moreno-Naranjo, Francesco Zinna, Lucas Frédéric, David B. Cordes, Aidan P. McKay, Matthew J. Fuchter, Xiaohong Zhang, Eli Zysman-Colman

**Affiliations:** a Organic Semiconductor Centre, EaStCHEM School of Chemistry, University of St Andrews St Andrews, Fife KY16 9ST UK eli.zysman-colman@st-andrews.ac.uk +44 1334 463808 +44 1334 463826; b Institute of Functional Nano & Soft Materials (FUNSOM), Joint International Research Laboratory of Carbon-Based Functional Materials and Devices, Soochow University Suzhou Jiangsu 215123 P. R. China; c Jiangsu Key Laboratory of Advanced Negative Carbon Technologies, Soochow University Suzhou Jiangsu 215123 P. R. China; d Department of Chemistry, Molecular Sciences Research Hub, Imperial College London, White City Campus London W12 0BZ UK m.fuchter@imperial.ac.uk; e Dipartimento di Chimica e Chimica Industriale, Università di Pisa 56124 Pisa Italy; f Université Paris-Saclay, ENS Paris-Saclay, CNRS, PPSM 91190 Gif-sur-Yvette France

## Abstract

Chiral multiresonant thermally activated delayed fluorescence (MR-TADF) materials show great potential as emitters in circularly polarized (CP) organic light-emitting diodes (CP-OLEDs) owing to their bright and narrowband CP emission. Here, two new chiral MR-TADF emitters *t*BuPh-BN and DPA-*t*BuPh-BN possessing intrinsically helical chirality have been synthesized and studied. The large steric interactions between the *tert*-butylphenyl groups not only induce the helical chirality but also provide a notable configurational stability to the enantiomers. Racemic mixtures of *t*BuPh-BN and DPA-*t*BuPh-BN show narrowband emission at 490 and 477 nm with full-width at half maximum (FWHM) of 25 and 28 nm and photoluminescence quantum yields, *Φ*_PL_, of 85 and 54% in toluene. The separated enantiomers of *t*BuPh-BN and DPA-*t*BuPh-BN show symmetric circularly polarized luminescence (CPL) with respective dissymmetry factors |*g*_PL_| values of 1.5 × 10^−3^ and 0.9 × 10^−3^. The hyperfluorescence organic light-emitting diodes (HF-OLEDs) with *t*BuPh-BN and DPA-*t*BuPh-BN acting as terminal emitters and 2,3,4,5,6-penta-(9*H*-carbazol-9-yl)benzonitrile (5CzBN) as their assistant dopant exhibited, respectively, maximum external quantum efficiencies (EQE_max_) of 20.9 and 15.9% at 492 and 480 nm with FWHM of 34 and 38 nm. This work demonstrates a strategy for developing intrinsically helically chiral MR-TADF emitters possessing significant configurational stability, which can be used in HF-OLEDs.

## Abstract 2

Helically chiral MR-TADF compounds *t*BuPh-BN and DPA-*t*BuPh-BN show narrowband circularly polarized luminescence that translates into high-performance hyperfluorescent organic light-emitting diodes.

## Graphical abstract

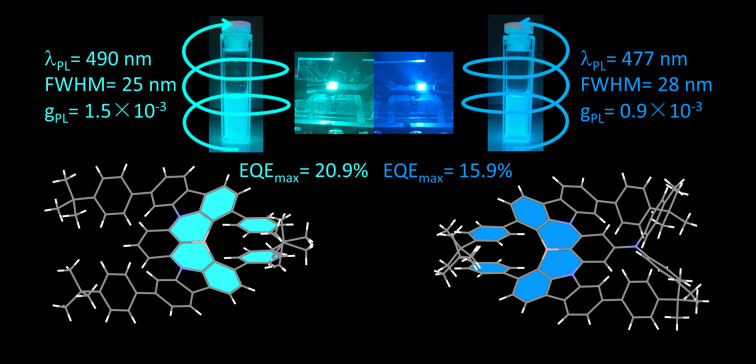

## Introduction

Chiral molecules emitting circularly polarized luminescence (CPL) have been widely investigated for their promising applications in three-dimensional (3D) displays, optical data storage and optical spintronics applications.^1–4^ In addition, this class of emitters has garnered significant interest as CPL offers a tantalizing solution to increasing the effective efficiency of organic light-emitting diodes (OLEDs)^5^ that contain anti-glare filters, which are required to improve the viewing contrast by limiting the reflection of external light.^6–8^ This requires materials to emit CPL selectively of one handedness. The propensity for CPL to possess a particular handedness is quantified by the dissymmetry factor *g*_PL_, which is described by eqn (1):1
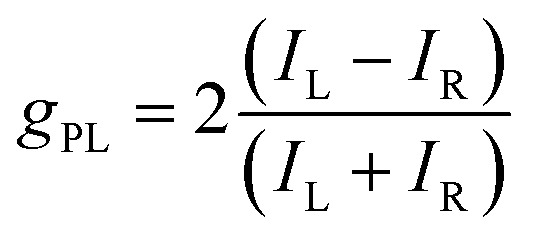
where *I*_L_ and *I*_R_ refer to the intensities of left- and right-handed light, respectively. Pure left- or right-handed light is obtained when *g*_PL_ reaches +2 or −2. For a chiral emissive material to be relevant for practical applications such as OLEDs, the |*g*_PL_| should be as high as possible,^9^ the photoluminescence quantum yield (*Φ*_PL_) should be high, and emission should be narrowband.

Organic thermally activated delayed fluorescence (TADF) materials have been widely explored as emitters in OLEDs over the past decade because of their ability to harvest both triplet and singlet excitons to produce light, leading to OLEDs with TADF emitters that can achieve 100% internal quantum efficiency (IQE).^10,11^ Multi-resonant TADF emitters, a sub-class of TADF materials, show characteristically narrowband emission and high *Φ*_PL_ due to their rigid structures and their possessing an emissive S_1_ state of short-range charge transfer (SRCT) character.^12^ Since the first reported example of a MR-TADF emitter by Hatakeyama and co-workers in 2016,^13^ now more than 250 examples of MR-TADF emitters for OLEDs have been reported, with many examples of devices showing maximum external quantum efficiencies (EQE_max_) of >30%.^14,15^ Of the reported MR-TADF emitters there are a small number that are chiral and CPL-active.

There are mainly two strategies for designing chiral MR-TADF emitters.^16^ The first one is chiral perturbation, which refers to introducing a peripheral chiral unit into an achiral MR-TADF structure that does not directly participate in the radiative S_1_–S_0_ transition. Li *et al.* reported the first two chiral MR-TADF emitters OBN-2CN-BN and OBN-4CN-BN containing (*R*)/(*S*)-octahydro-binaphthol ((*R*)/(*S*)-OBN) units (Fig. S40, Table S6†).^17^ These two compounds emit at *λ*_PL_ of 498 and 510 nm (full-width at half maximum, FWHM of 32 and 35 nm), have *Φ*_PL_ of 95 and 90% and |*g*_PL_| values of 9.1 × 10^−4^ and 1.04 × 10^−3^ in 3 wt% doped films in PhCzBCz, respectively. Although materials based on chiral perturbation are generally easy to synthesize and do not need enantiomer separation as the chiral unit that is incorporated is generally already enantiopure, they tend to show weak CPL signals.^16,18^ The second strategy involves the design of MR-TADF emitters that possess an intrinsically chiral skeleton.^19,20^ To date, there are only a few examples of chiral MR-TADF emitters based on this strategy. They have either point,^21–24^ axial,^25–28^ planar,^29,30^ or helically^31–43^ chiral skeletons (Fig. S40, Table S6†). Of particular note, Guo *et al.* developed a large helicene-based chiral MR-TADF emitter BN[9]H, which emits at *λ*_PL_ of 578 nm (FWHM of 47 nm) and has a *Φ*_PL_ of 98% in toluene.^42^ The |*g*_PL_| is 5.8 × 10^−3^ in toluene, which is the highest value among all the reported chiral MR-TADF emitters to date. The CP-OLEDs with *P*-BN[9]H emitted at *λ*_EL_ of 580 nm and showed an EQE_max_ of 35.4% and a low efficiency roll-off (EQE_100_ of 33.1%). The *g*_EL_ value of 6.2 × 10^−3^ was of similar magnitude to the |*g*_PL_|.

**Fig. 1:**
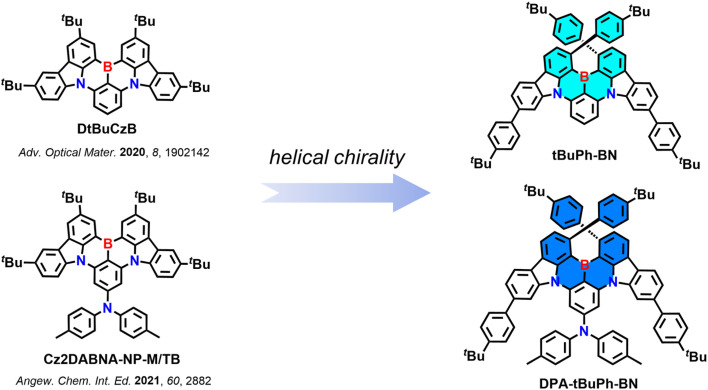
Molecular design of *t*BuPh-BN and DPA-*t*BuPh-BN.

Here, we report two new chiral MR-TADF emitters *t*BuPh-BN and DPA-*t*BuPh-BN (Fig. 1) based on the CzBN core of DtBuCzB,^44^ a compound that shows narrowband emission at 481 nm (FWHM of 22 nm) and high *Φ*_PL_ of 91% in toluene. The *tert*-butylphenyl groups are introduced to induce helical chirality and the large steric hindrance between them is hypothesized to inhibit the racemization between the two helically chiral enantiomers. A diphenylamine (DPA) group is introduced to the *para* position of the boron-substituted phenyl ring in DPA-*t*BuPh-BN to explore the effect that peripheral units have on the optoelectronic properties of these chiral MR-TADF compounds. *t*BuPh-BN and DPA-*t*BuPh-BN exhibit narrow sky-blue emission at 490 nm (FWHM of 25 nm) and 477 nm (FWHM of 28 nm) and have *Φ*_PL_ of 85 and 54% in toluene, respectively. Due to their large Δ*E*_ST_ values of 0.36 and 0.43 eV for *t*BuPh-BN and DPA-*t*BuPh-BN, they show long delayed lifetimes, *τ*_d_, of 41 and 60 ms in 2 wt% doped films in SF3-TRZ, respectively. The resolved enantiomers of *t*BuPh-BN and DPA-*t*BuPh-BN show symmetric CPL, with |*g*_PL_| values of 1.5 × 10^−3^ and 0.9 × 10^−3^, respectively. Hyperfluorescence (HF) OLEDs using 2,3,4,5,6-penta-(9*H*-carbazol-9-yl)benzonitrile (5CzBN) as the TADF assistant dopant and with *t*BuPh-BN and DPA-*t*BuPh-BN as the terminal emitters showed narrowband emission at *λ*_EL_ of 492 and 480 nm (FWHM of 34 and 38 nm) and EQE_max_ of 20.9 and 15.9%, respectively.

## Results & discussion

### Synthesis

**Fig. 2:**
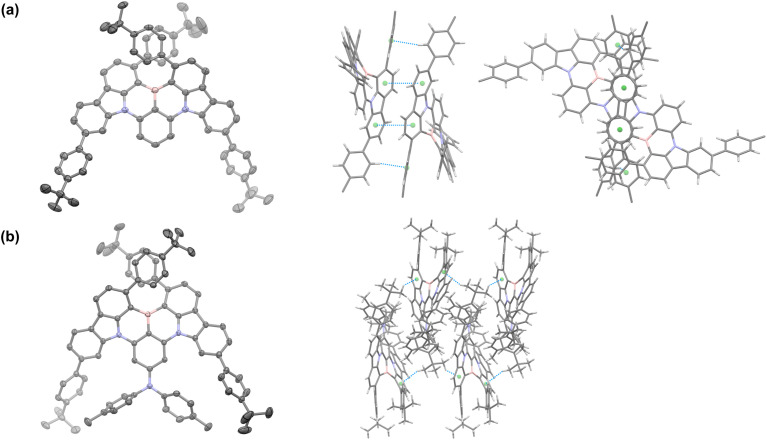
Thermal ellipsoid plot of the structures and intermolecular interactions of (a) *t*BuPh-BN and (b) DPA-*t*BuPh-BN. Ellipsoids are displayed at the 50% probability level, solvent molecules, minor components of disorder and hydrogens are omitted for clarity.

The synthesis of *t*BuPh-BN and DPA-*t*BuPh-BN is outlined in Fig. S1.† The intermediate 1 was prepared under Suzuki–Miyaura cross-coupling conditions in 64% yield, which was then reacted through a nucleophilic aromatic substitution to afford 2 and 3 in 74 and 97% yield, respectively, while compound 4 was prepared by a palladium-catalyzed Buchwald–Hartwig amination between compound 3 and di-*p*-tolylamine in 60% yield. *t*BuPh-BN and DPA-*t*BuPh-BN were obtained from 2 and 4, respectively, following an electrophilic borylation in 17 and 11% yield, respectively. The relatively low yields can be mainly attributed to the large steric hindrance around the boron atom. Their identity and purity were characterized using melting point determination, ^1^H and ^13^C nuclear magnetic resonance (NMR) spectroscopy, high-resolution mass spectrometry (HRMS), high-performance liquid chromatography (HPLC), and elemental analysis (EA) (Fig. S2–S23†). The structures of racemic *t*BuPh-BN and DPA-*t*BuPh-BN were confirmed by single crystal X-ray diffraction analysis (Fig. 2, Table S1†). X-ray quality crystals were obtained from slow evaporation of THF:MeOH solutions of *t*BuPh-BN and DPA-*t*BuPh-BN. The structure of DPA-*t*BuPh-BN was found to be enantiopure and showing just the *M*-enantiomer, despite the crystals having been grown from the racemic mixture from synthesis. Both emitters have conformations distorted away from planarity due to the large steric hindrance between the two proximal *tert*-butylphenyl groups. This forces a twist onto the core of the molecule, carbazole groups being inclined at 40.25(5) and 37.92(3)° for *t*BuPh-BN and DPA-*t*BuPh-BN, respectively, endowing them with helical chirality. Both structures showed only weak interactions between adjacent molecules. Molecules of *t*BuPh-BN interacted *via* a combination of mutually supporting π–π and edge-to-face CH⋯π interactions, with centroid···centroid distances of 3.787(2) Å, and H···centroid distance of 2.68 Å (corresponding C···centroid separation of 3.572(4) Å). These interactions form discrete dimers of *t*BuPh-BN. In contrast, molecules of DPA-*t*BuPh-BN display two sets of CH_methyl_⋯π interactions between, with H···centroid distances of 2.70 and 2.78 Å (corresponding C···centroid separations of 3.642(3) and 3.411(3) Å). These interactions in combination led to the formation of weakly interacting two-dimensional sheet structures. The enantiomers of *t*BuPh-BN and DPA-*t*BuPh-BN were separated by chiral HPLC, to afford ee. 99% for (*P*)-*t*BuPh-BN, 97% for (*M*)-*t*BuPh-BN, 99% for (*P*)-DPA-*t*BuPh-BN and 99% for (*M*)-DPA-*t*BuPh-BN (Fig. S24 and S25†). The absolute configurations (*P* and *M*) here were tentatively determined by comparing the measured and simulated circular dichroism (CD) spectra in toluene solution (Fig. S26†). *t*BuPh-BN and DPA-*t*BuPh-BN are thermally stable and have high thermal decomposition temperatures (*T*_d_, corresponding to 5% weight loss) of 488 and 493 °C, respectively. No glass transition temperature was observed in the differential scanning calorimetry (DSC) measurement of either compound (Fig. S27†).

### Theoretical calculations

The optimized ground-state geometries of *t*BuPh-BN and DPA-*t*BuPh-BN were calculated using Density Functional Theory (DFT) at the PBE0/6-31G(d,p) level (Fig. S28†).^45,46^ The highest occupied molecular orbital (HOMO) and lowest unoccupied molecular orbital (LUMO) of *t*BuPh-BN are both located on the CzBN core. The calculated HOMO and LUMO energies of *t*BuPh-BN are −5.43/−1.78 eV and the energy gap (Δ*E*) is 3.65 eV. The slightly deeper HOMO and LUMO levels compared to those of DtBuCzB (HOMO = −5.28 eV, LUMO = −1.64 eV)^44^ may originate from an inductively electron-withdrawing effect from the *tert*-butylphenyl groups. By contrast, the HOMO of DPA-*t*BuPh-BN is localized on the electron-donating DPA moiety, and this leads to a destabilization of the both the HOMO and LUMO levels at −5.23 and −1.66 eV, respectively.

**Fig. 3:**
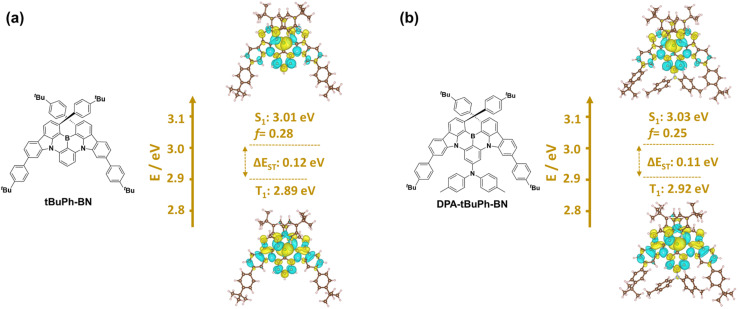
Difference density plots and energies of S_1_ and T_1_ calculated in the gas phase at SCS-(ADC)2/cc-pVDZ level, respectively, for (a) *t*BuPh-BN and (b) DPA-*t*BuPh-BN (Blue indicates an area of decreased electron density while yellow indicates increased electronic density between the ground and excited states).

The excited-state energies and difference densities were calculated using Spin-Component Scaling second-order algebraic diagrammatic construction (SCS-(ADC)2/cc-pVDZ).^47,48^ As shown in Fig. 3, both emitters have difference density patterns for both the S_1_ and T_1_ states that are reminiscent of SRCT states localized on the CzBN core. *t*BuPh-BN and DPA-*t*BuPh-BN have S_1_/T_1_ energies of 3.01/2.89 eV and 3.03/2.92 eV, respectively, and the corresponding Δ*E*_ST_ values are similar at 0.12 and 0.11 eV, respectively. Spin–orbit coupling matrix elements (SOCME) were also calculated based on the optimized T_1_ geometries at the PBE0/6-31G(d,p) level (Fig. S28†). *t*BuPh-BN and DPA-*t*BuPh-BN have similar SOCME values of 0.17, 0.35, 0.23 and 0.20, 0.33, 0.19 cm^−1^ for the S_1_–T_1_, S_1_–T_2_, S_1_–T_3_ transitions, respectively. The larger SOCME and smaller energy gaps between S_1_ and T_2_ for both *t*BuPh-BN and DPA-*t*BuPh-BN indicate that RISC may proceed *via* T_2_ to S_1_.^49–52^

### Optoelectronic properties

The HOMO and LUMO values of *t*BuPh-BN and DPA-*t*BuPh-BN were extrapolated from the electrochemistry measurements in degassed dichloromethane with 0.1 M [^*n*^Bu_4_N]PF_6_ as the supporting electrolyte and Fc/Fc^+^ as the internal reference (0.46 V *vs.* SCE)^53^ (Fig. S29†). The electrochemical data are summarized in Table S2.† The cyclic voltammograms of both *t*BuPh-BN and DPA-*t*BuPh-BN show reversible oxidation waves. *t*BuPh-BN has an oxidation potential, *E*_ox_, of 1.19 V *vs.* SCE, obtained from the peak of the differential pulse voltammogram. The corresponding HOMO energy is −5.53 eV, which is close to the HOMO of DtBuCzB (DtCzB) (−5.40 eV),^44,54^ indicating that it is localized on the CzBN core. By contrast, the *E*_ox_ of DPA-*t*BuPh-BN is cathodically shifted to 0.97 V *vs.* SCE (HOMO of −5.31 eV). This value is very similar to the HOMO of *t*DPA-DtCzB, which contains a 4,4′-di-*tert*-butylphenylamine donor (HOMO of −5.24 eV), indicating that oxidation occurs at the amine group.^54^ Both *t*BuPh-BN and DPA-*t*BuPh-BN show reversible reduction waves with reduction potentials (*E*_red_) of −1.77 and −1.85 V, respectively; the corresponding LUMO energies are −2.57 and −2.49 eV. The small differences in LUMO levels compared to those of DtCzB (−2.61 eV) and *t*DPA-DtCzB (−2.38 eV)^54^ indicate that the LUMO in both *t*BuPh-BN and DPA-*t*BuPh-BN is situated on the DtCzB core and that the amine donor group in DPA-*t*BuPh-BN promotes a destabilization of the LUMO level compared to *t*BuPh-BN. The corresponding Δ*E* values are calculated to be 2.96 and 2.82 eV, respectively. The trends in orbital energies align well with the DFT calculations (−5.43/−1.78 eV and −5.23/−1.66 eV, respectively).

**Fig. 4:**
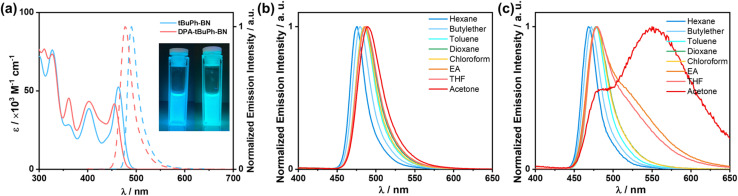
(a) UV-vis absorption spectra and PL spectra of *t*BuPh-BN and DPA-*t*BuPh-BN in toluene at 300 K; inset: photos of *t*BuPh-BN (right) and DPA-*t*BuPh-BN (left) in toluene excited at 365 nm. PL spectra of (b) *t*BuPh-BN and (c) DPA-*t*BuPh-BN in different solvents at 300 K. (*λ*_exc_ = 340 nm).

The ultraviolet-visible (UV-vis) absorption and photoluminescence (PL) properties of the two emitters were first investigated in toluene at room temperature (Fig. 4a). The absorption spectra of *t*BuPh-BN and DPA-*t*BuPh-BN show low-energy bands at 463 and 455 nm, respectively, which are attributed to the SRCT S_0_–S_1_ transitions and are close to that of DtBuCzB (*λ*_abs_ of 467 nm).^44^ The higher molar absorptivity (*ε*) of this SRCT band in *t*BuPh-BN (5.2 × 10^4^ M^−1^ cm^−1^) than that in DPA-*t*BuPh-BN (4.2 × 10^4^ M^−1^ cm^−1^) is correlated to the higher calculated oscillator strength of the S_0_–S_1_ transitions (0.28 for *t*BuPh-BN and 0.25 for DPA-*t*BuPh-BN). *t*BuPh-BN is a narrowband emitter, with its photoluminescence peaking at *λ*_PL_ of 490 nm and having a FWHM, of 25 nm (0.13 eV). The emission properties are comparable to those of DtBuCzB (*λ*_PL_ of 481 nm, FWHM of 22 nm in toluene),^44^ which illustrates that the nature of the S_1_ state is the same in these compounds. By contrast for DPA-*t*BuPh-BN, the DPA donor positioned *para* to the boron atom reduces its electron-withdrawing character and weakens the SRCT transition localized on the CzBN core,^55^ resulting in a blue-shifted emission band at 477 nm, which also aligns with the slightly higher calculated S_1_ energy (3.03 eV) compared to that of *t*BuPh-BN (3.01 eV) (Fig. 3). The slightly broader FWHM of 28 nm/0.15 eV can be attributed to the larger degree of geometry relaxation on the DPA group between ground and excited states. The photophysics of DPA-*t*BuPh-BN is reminiscent of that of tDPA-DtCzB (*λ*_PL_ of 470 nm and FWHM of 21 nm in toluene).^54^ The larger Stokes shifts of *t*BuPh-BN (27 nm) and DPA-*t*BuPh-BN (22 nm) compared to that of DtBuCzB (14 nm)^44^ and *t*DPA-DtCzB (14 nm)^54^ indicate a larger degree of structural relaxation between ground and excited states due to the introduction of the *tert*-butylphenyl groups. *t*BuPh-BN and DPA-*t*BuPh-BN have contrasting *Φ*_PL_ values in degassed toluene of 85 and 54%, respectively. A solvatochromic study on the impact of solvent polarity on the PL spectra of *t*BuPh-BN and DPA-*t*BuPh-BN is shown in Fig. 4b and c. *t*BuPh-BN shows a very small degree of positive solvatochromism, which confirms that the SRCT character of the S_1_ state is conserved across all these solvents. By contrast, there are two regimes of behavior for DPA-*t*BuPh-BN, where in low polarity solvents emission from the SRCT state dominates but in higher polarity solvents dual emission is observed. This is due to emission originating from both the SRCT state and a long-range charge-transfer state (LRCT) from the strong DPA donor to the weak CzBN acceptor as this latter state becomes increasingly stabilized in higher polarity solvents, a behavior that was observed previously in donor-substituted DiKTa derivatives.^56^ The unchanging PL spectra of DPA-*t*BuPh-BN in acetone under different concentrations confirm that the latter emission band is not from aggregates (Fig. S30†).

The steady-state PL (SS PL) and phosphorescence (Ph) spectra were measured in 2-MeTHF at 77 K to determine the singlet and triplet energies and, thus, the Δ*E*_ST_ values of *t*BuPh-BN and DPA-*t*BuPh-BN (Fig. S31†). The singlet and triplet energies were determined from the onsets of the SS PL and Ph spectra, respectively. The S_1_/T_1_ energies for *t*BuPh-BN and DPA-*t*BuPh-BN are 2.60/2.24 and 2.71/2.28 eV, respectively, resulting in Δ*E*_ST_ values of 0.36 and 0.43 eV. Although their S_1_ energies are similar to those of DtBuCzB (2.66 eV) and *t*DPA-DtCzB (2.72 eV), their T_1_ energies are much lower (DtBuCzB (2.53 eV) and *t*DPA-DtCzB (2.61 eV)), which leads to much larger Δ*E*_ST_ values than the 0.13 eV for DtBuCzB and 0.11 eV for *t*DPA-DtCzB.^44,54^ To explore the origin for the divergent T_1_ energies and Δ*E*_ST_ values compared to the literature reference compounds, the S_1_ and T_1_ energies of the fragments, 2,7-diphenyl-9*H*-carbazole (2,7-PhCz) and 3,6-diphenyl-9*H*-carbazole (3,6-PhCz), were calculated using time-dependent DFT within the Tamm–Dancoff approximation (Fig. S32†). Both 2,7-PhCz and 3,6-PhCz have almost the same S_1_ energies of around 4 eV; however, as the electron density of T_1_ in 2,7-PhCz is delocalized across the whole molecule, this leads to a much-stabilized T_1_ energy of 3.04 eV compared to that of 3,6-PhCz (3.33 eV). Thus, this explains the much larger Δ*E*_ST_ values for *t*BuPh-BN and DPA-*t*BuPh-BN as there is a much more stabilized locally-excited state situated on the 2,7-PhCz donor groups. The time-resolved PL decays in degassed toluene are shown in Fig. S31c.† The emission of both *t*BuPh-BN and DPA-*t*BuPh-BN decay with monoexponential kinetics with short lifetimes (*τ*_PL_) of 5.8 and 4.4 ns, respectively. Due to the large Δ*E*_ST_ values and the competing non-radiative decay processes with ISC/RISC,^57^ no delayed emission was observed in toluene solution.

**Fig. 5:**
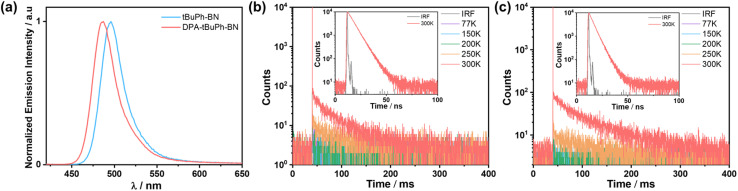
(a) Steady-state PL spectra at 300 K of 2 wt% doped film of emitters in SF3-TRZ (*λ*_exc_ = 340 nm). Temperature-dependent time-resolved PL decays of in 2 wt% doped film of (b) *t*BuPh-BN and (c) DPA-*t*BuPh-BN in SF3-TRZ (inset figures are PL decays of the prompt components at 300 K) (*λ*_exc_ = 375 nm).

The photophysical properties of thin films were then investigated. The host 2-(9,9′-spirobi[fluoren]-3-yl)-4,6-diphenyl-1,3,5-triazine (SF3-TRZ) was chosen because of its suitably high triplet energy (*E*_T_ = 2.80 eV) and ability to balance charge transport in a HF OLED using 5CzBN as the assistant dopant.^58^ The absolute *Φ*_PL_ values at different doping concentrations were measured under a nitrogen atmosphere (Table S3†). Both compounds show a propensity to aggregate that is reflected in their lower *Φ*_PL_ (67% to 42% for *t*BuPh-BN and 47% to 30% for DPA-*t*BuPh-BN) and slightly broader and red-shifted emission (490 to 496 nm for *t*BuPh-BN and 484 to 491 nm for DPA-*t*BuPh-BN) of the 1, 2, 4 and 10 wt% doped films compared to the results from toluene solution (*Φ*_PL_ of 85% and 54%, *λ*_PL_ of 490 and 477 nm, respectively) (Fig. S33†). The 2 wt% doped films of *t*BuPh-BN and DPA-*t*BuPh-BN in SF3-TRZ emit at *λ*_PL_ of 496 and 487 nm (FWHMs of 31 nm/0.16 eV and 31 nm/0.16 eV), respectively (Fig. 5a). At 300 K under vacuum, the time-resolved PL decays of *t*BuPh-BN and DPA-*t*BuPh-BN show biexponential kinetics, with a dominant prompt lifetime, *τ*_p_, of 5.9 and 4.6 ns and long *τ*_d_ of 41 and 60 ms, respectively (Table 1). The largely decreased delayed components to the lifetime of *t*BuPh-BN with an increase in the doping concentration from 0.5 to 2 wt% doped film and the neat film indicate that the delayed emission originates from the isolated molecule rather than from aggregates (Fig. S34†). An analysis of the temperature-dependent time-resolved PL decays under vacuum reveal an increasing intensity of the delayed emission with increasing temperature that is characteristic of TADF (Fig. 5b and c).

**Table 1:** Photophysical data of *t*BuPh-BN and DPA-*t*BuPh-BN

Compound	*λ*_abs_ a/nm	*λ*_PL_ b (FWHM)/nm	Δ*E*_ST_^c^/eV	|*g*_PL_|^d^	*Φ*_PL_ e/%	*λ*_PL_ f (FWHM)/nm	*τ*_p_ g/ns	*τ*_d_ g/ms
*t*BuPh-BN	463	490 (25)	0.36	1.5 × 10^−3^	85	496 (31)	5.9	41
DPA-*t*BuPh-BN	455	477 (28)	0.43	0.9 × 10^−3^	54	487 (31)	4.6	60

a In toluene.

b In toluene at room temperature, *λ*_exc_ = 340 nm.

c In 2-MeTHF at 77 K, *λ*_exc_ = 340 nm, Δ*E*_ST_ = *E*(S_1_) − *E*(T_1_).

d In THF at room temperature, *λ*_exc_ = 365 nm.

e In degassed toluene at room temperature, *λ*_exc_ = 350 nm.

f In 2 wt% doped films in SF3-TRZ at 300 K under vacuum, *λ*_exc_ = 340 nm.

g In 2 wt% doped films in SF3-TRZ at 300 K under vacuum, *λ*_exc_ = 375 nm.

**Fig. 6:**
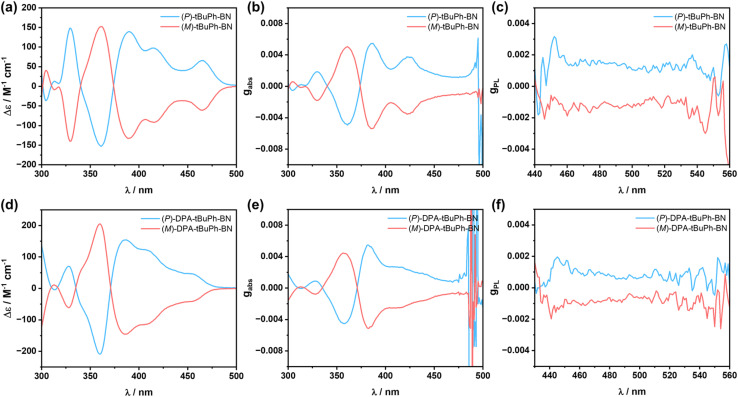
(a) CD spectra and (b) *g*_abs_ values of *t*BuPh-BN recorded in 5 × 10^−6^ mol L^−1^ toluene solution. (c) *g*_PL_ values of *t*BuPh-BN recorded in 10^−5^ mol L^−1^ THF solution. (d) CD spectra and (e) *g*_abs_ values of DPA-*t*BuPh-BN recorded in 5 × 10^−6^ mol L^−1^ toluene solution. (f) *g*_PL_ values of DPA-*t*BuPh-BN recorded in 10^−5^ mol L^−1^ THF solution.

The chiroptical properties of (*P*)/(*M*)-*t*BuPh-BN and (*P*)/(*M*)-DPA-*t*BuPh-BN were investigated in solution. As shown in Fig. 6a and d, both emitters display typical mirror image CD spectra between *P* and *M* enantiomers. The maximum absorption dissymmetry factor *g*_abs_ values are +5.5 × 10^−3^ and −5.4 × 10^−3^ at 385 nm for (*P*)/(*M*)-*t*BuPh-BN and +5.5 × 10^−3^ and −5.1 × 10^−3^ at 382 nm for (*P*)/(*M*)-DPA-*t*BuPh-BN, respectively. Both emitters also show mirror symmetric CPL spectra in THF solutions (Fig. 6c and f) with *g*_PL_ values of +1.5 × 10^−3^ and −1.3 × 10^−3^ for (*P*)/(*M*)-*t*BuPh-BN and +0.9 × 10^−3^ and −0.8 × 10^−3^ for (*P*)/(*M*)-DPA-*t*BuPh-BN, respectively. To better understand the origin of the differing *g*_PL_ values between *t*BuPh-BN and DPA-*t*BuPh-BN, TDA-DFT calculations starting from the optimized S_1_ geometries were performed to predict the *g*_PL_ values in THF (Table S4†). The M062X/6-31G(d,p) level of theory was chosen here because it accurately predicts the SRCT character of the S_1_ state of DPA-*t*BuPh-BN, while PBE0/6-31G(d,p) calculations incorrectly predict that this state should have LRCT character (Fig. S35†). The calculated *g*_PL_ values of (*M*)-*t*BuPh-BN and (*M*)-DPA-*t*BuPh-BN are −1.6 × 10^−3^ and −0.67 × 10^−3^, respectively, which align well with the experimental values. Both emitters have similar predicted electric transition dipole moment |*μ*| and angles between *μ* and the magnetic transition dipole moment *m*. The lower *g*_PL_ value of (*M*)-DPA-*t*BuPh-BN therefore mainly originates from the lower magnitude of |*m*|. CPL brightness (*B*_CPL_) was also estimated as *B*_CPL_ = *ε* × *Φ*_PL_ × *g*_PL_/2.^59^ The *ε* and *Φ*_PL_ values of *t*BuPh-BN and DPA-*t*BuPh-BN in THF are 3.9 × 10^4^ M^−1^ cm^−1^, 66%, and 3.8 × 10^4^ M^−1^ cm^−1^, 49%, respectively. The calculated B_CPL_ values of (*P*)-*t*BuPh-BN and (*P*)-DPA-*t*BuPh-BN are 19.3 and 8.4 M^−1^ cm^−1^, respectively. These values are at the high end of those calculated for helicenes^59^ and comparable to the reported high-performance chiral MR-TADF emitters,^35,40,41,43^ illustrating that *t*BuPh-BN and DPA-*t*BuPh-BN are efficient CPL-active TADF helicenes. To explore the stereochemical stability of *t*BuPh-BN and DPA-*t*BuPh-BN, the racemization energy barrier of the helicene moiety was calculated using DFT at the M062X/6-31G(d,p) level of theory (Fig. S36†). The calculated high energy barrier of Δ*G*^‡^ = 48.28 kcal mol^−1^ enables the chiral separation of *t*BuPh-BN and DPA-*t*BuPh-BN.^33,35,41,42^ In addition, no racemization was observed at 90 °C in toluene over more than 5 hours as the CD signals of (*P*)-*t*BuPh-BN and (*P*)-DPA-*t*BuPh-BN remained constant (Fig. S37†).^38^ Both computational and experimental results indicate that the large steric hindrance between the top two *tert*-butylphenyl groups provide a notable stereochemical stability to the enantiomers.

### Organic light-emitting diodes

**Fig. 7:**
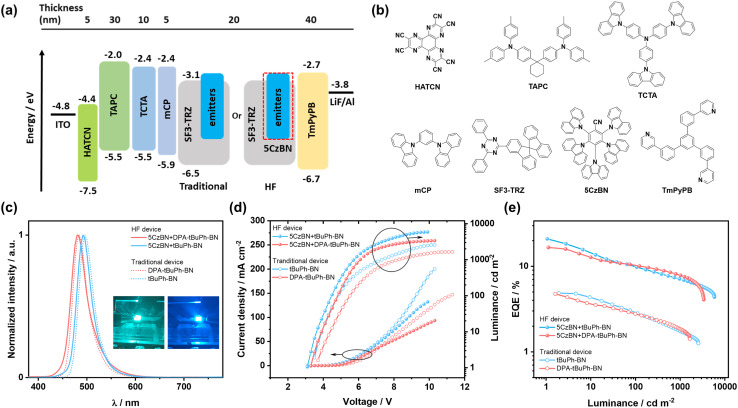
(a) Schematic of the device stack of the OLEDs; (b) chemical structures of the organic layers; (c) EL spectra recorded at 5 V, the insets are the photograph images of the HF devices for *t*BuPh-BN (left) and DPA-tBuPh-BN (right); (d) *JVL* characteristics; (e) EQE *vs.* luminance for the OLEDs.

We next fabricated vacuum-deposited OLEDs with *t*BuPh-BN and DPA-*t*BuPh-BN using a device structure of indium tin oxide (ITO)/1,4,5,8,9,11-hexaazatriphenylenehexacarbonitrile (HATCN, 5 nm)/1,1-bis[(di-4-tolylamino)phenyl]cyclohexane (TAPC, 30 nm)/tris(4-carbazoyl-9-ylphenyl)amine (TCTA, 10 nm)/mCP (5 nm)/emitting layer (EML) (20 nm)/1,3,5-tris(3-pyridyl-3-phenyl)benzene (TmPyPB, 40 nm)/lithium fluoride (LiF, 1 nm)/aluminum (Al, 100 nm). Here, HATCN was used as the hole injection layer, TAPC and TCTA as the hole transporting layers, mCP as an exciton blocking layer, TmPyPB as an electron transporting layer, and LiF was used to reduce the work function of the top Al electrode. The OLED device stack and the chemical structures of the organic layers are shown in Fig. 7a and b. The device data are summarized in Table 2.

**Table 2:** Device data of *t*BuPh-BN and DPA-*t*BuPh-BN

Traditional device^a^	*V*_on_/V	*λ*_EL_ c (FWHM)/nm	*L*_max_/cd m^−2^	EQE_max/100/1000_/%	CIE^c^ (*x*, *y*)
*t*BuPh-BN	3.5	496 (34)	2560	4.9/2.8/1.7	0.10, 0.50
DPA-*t*BuPh-BN	3.7	484 (34)	1650	4.8/2.8/1.8	0.11, 0.31

**HF device** b					
*t*BuPh-BN	3.1	492 (34)	5850	20.9/9.8/7.1	0.11, 0.41
DPA-*t*BuPh-BN	3.3	480 (38)	3370	15.9/10.1/7.8	0.13, 0.29

a 4 wt% emitters in SF3-TRZ as EMLs.

b 2 wt% emitters and 12 wt% 5CzBN in SF3-TRZ as EMLs.

c Recorded at 5 V.

We first fabricated OLEDs with a higher concentration of 4 wt% emitters in SF3-TRZ as the EML as it was hypothesized that a higher concentration of emitter would contribute to improve the charge balance and enlarge the exciton recombination zone. Electroluminescence (EL) spectra, current density–voltage–luminance (*JVL*) curves and EQE *vs.* luminance curves are shown in Fig. 7c–e. *t*BuPh-BN and DPA-*t*BuPh-BN emitted narrowband green and sky-blue light at *λ*_EL_ of 496 and 484 nm, each having FWHM of 34 nm; the EL spectra are close to the corresponding PL spectra (Fig. 5). Due in part to their long delayed lifetimes the devices with *t*BuPh-BN and DPA-*t*BuPh-BN showed low EQE_max_ of 4.9 and 4.8% and severe efficiency roll-off (EQE_100_ of both 2.8% and EQE_1000_ of 1.7 and 1.8%, respectively). The Commission International de l'Éclairage, CIE, coordinates for the devices with *t*BuPh-BN and DPA-*t*BuPh-BN were (0.10, 0.50) and (0.11, 0.31). The OLEDs with 2 wt% emitters were also fabricated for comparison. Due to an incomplete energy transfer from the host to the emitter, the device with DPA-*t*BuPh-BN exhibited another blue emission band at around 420 nm, originating from emission from SF3-TRZ (Fig. S37†). When compared to the OLEDs with 4 wt% emitters, although the devices with 2 wt% of *t*BuPh-BN and DPA-*t*BuPh-BN showed bluer emission at *λ*_PL_ of 492 and 480 nm [CIE coordinates of (0.10, 0.45) and (0.12, 0.22)] due to the lower contribution to the emission from aggregates, their maximum luminance (*L*_max_) (2217 and 990 cd m^−2^) and EQE_max_ (4.6% for both devices) were decreased. This may arise from a limited exciton recombination zone in the EML of these devices. The performance and data are summarized in Table S5.†

In a bid to improve the device performance and given the narrowband emission and high *Φ*_PL_ values for *t*BuPh-BN and DPA-*t*BuPh-BN, HF OLEDs were then fabricated (Fig. 7a). 5CzBN was used as the TADF assistant dopant because of its high *Φ*_PL_ of 70%, fast *k*_RISC_ of 1.13 × 10^5^ s^−1^, and a strong overlap between the absorption spectra of *t*BuPh-BN and DPA-*t*BuPh-BN and the PL spectrum of 5CzBN in toluene (Fig. S39a†).^60^ An optimized doping ratio of 2 wt% emitters: 12 wt% 5CzBN: 86 wt% SF3-TRZ was identified to be used as the EML as this formulation minimized the chance for triplet excitons from the host to transfer to the emitters and permitted an efficient Förster resonance energy transfer from 5CzBN to the emitters (Fig. S39b and c†) while conserving their narrowband emission. As shown in Fig. 7, the HF devices with *t*BuPh-BN and DPA-*t*BuPh-BN showed narrowband emission at *λ*_PL_ of 492 and 480 nm and FWHMs of 34 nm (0.17 eV) and 38 nm (0.20 eV). The corresponding CIE coordinates were (0.11, 0.41) and (0.13, 0.29), respectively, which are similar to those of the 2 wt% emitter OLEDs (Fig. S38, Table S5†). The turn-on voltages (*V*_on_) were reduced from 3.5 and 3.7 V to 3.1 and 3.3 V and the EQE_max_ values were significantly improved to 20.9 and 15.9% for the HF devices with *t*BuPh-BN and DPA-*t*BuPh-BN, respectively. The relatively higher EQE_max_ for the HF OLED with *t*BuPh-BN can be attributed to the higher *Φ*_PL_ of the emitter and the larger overlap between the absorption spectra of the MR-TADF emitters and the PL spectrum of 5CzBN (Spectral overlap integrals^61,62^ were estimated to be 6.0 × 10^13^ M^−1^ cm^−1^ nm^4^ for *t*BuPh-BN and 4.2 × 10^13^ M^−1^ cm^−1^ nm^4^ for DPA-*t*BuPh-BN). Despite their higher EQE_max_, the efficiency roll-off of the HF devices remained severe (EQE_100_ of 9.8 and 10.1% and EQE_1000_ of 7.1 and 7.8% for the HF devices with *t*BuPh-BN and DPA-*t*BuPh-BN, respectively). Two possible explanations are that the competing processes of direct exciton recombination on the emitters or the short-range Dexter energy transfer from 5CzBN to that of the emitters are still strong.^54^ The accumulation of triplet excitons would increase the probability of bimolecular excitonic quenching processes that manifest in severe efficiency roll-off in the devices.

## Conclusions

Two new MR-TADF emitters *t*BuPh-BN and DPA-*t*BuPh-BN possessing intrinsically helical chirality have been designed and synthesized. The SCS-(ADC)2 calculations and the narrowband emission at 490 and 477 nm (FWHM of 25 and 28 nm) for *t*BuPh-BN and DPA-*t*BuPh-BN, respectively, in toluene corroborate an emission from a SRCT excited state. The large steric hindrance between two of the *tert*-butylphenyl groups provides both molecules with significant thermal stability towards racemization, resulting in configurational stability even at 90 °C. As a result, the separated enantiomers of *t*BuPh-BN and DPA-*t*BuPh-BN show symmetric CD and CPL spectra, with PL dissymmetry factor values, |*g*_PL_|, of 1.5 × 10^−3^ and 0.9 × 10^−3^, respectively. Finally, the optimized HF-OLEDs with *t*BuPh-BN and DPA-*t*BuPh-BN as the terminal emitter achieved narrowband sky-blue emission with EQE_max_ of 20.9 and 15.9%, respectively. This study illustrates the effect of the addition of sterically bulky groups at the 2 and 7 positions of carbazole on the properties of derived MR-TADF emitters on both the photophysical and chiroptical performance.

## Data availability

The research data supporting this publication can be accessed at https://doi.org/10.17630/3dbc2b86-2c76-464e-8423-cd5c95247a07.

## Author contributions

Jingxiang Wang performed the synthesis, the theoretical calculations, the optoelectronic characterization and wrote the original manuscript. The molecules were designed by Dr Lucas Frédéric. The crystal structures were solved by Dr Aidan P. McKay and Dr David B. Cordes. The OLEDs were fabricated by Dr Dongyang Chen. The chiroptical properties were measured by Juan Manuel Moreno-Naranjo and Dr Francesco Zinna. Prof. Matthew J. Fuchter, Prof. Xiaohong Zhang and Prof. Eli Zysman-Colman were responsible for supervision and financing the project and revised the manuscript. Prof. Eli Zysman-Colman managed the project.

## Conflicts of interest

The authors declare no conflict of interest.

## Supplementary Material

SC-OLF-D4SC03478C-s001

SC-OLF-D4SC03478C-s002

SC-OLF-D4SC03478C-s003
