# Supplementary material for: Helically chiral multiresonant thermally activated delayed fluorescent emitters and their use in hyperfluorescent organic light-emitting diodes
Source: Chem Sci. 2024 Sep 18;15(41):16917–27. doi: 10.1039/d4sc03478c (PMC11420764; doi:10.1039/d4sc03478c)
Supplement: SC-015-D4SC03478C-s002 [file SC-015-D4SC03478C-s002.pdf]

Supplementary Information (SI) for Chemical Science.
This journal is © The Royal Society of Chemistry 2024

Helically chiral multiresonant thermally activated delayed fluorescent emitters and their use in hyperfluorescent organic light-emitting diodes

Jingxiang Wang[a‡], *Dongyang Chen*[a,b,c‡], *Juan Manuel Moreno-Naranjo*[d], *Francesco Zinna*[e], *Lucas Frédéric*[f], *David B. Cordes*[a], *Aidan P. McKay*[a], *Matthew J. Fuchter*[d*], *Xiaohong Zhang*[b,c*] *and Eli Zysman-Colman*[a*]

[a] Organic Semiconductor Centre, EaStCHEM School of Chemistry, University of St Andrews, St Andrews, Fife, UK, KY16 9ST, Fax: +44-1334 463808; Tel: +44-1334 463826; E-mail: eli.zysman-colman@st-andrews.ac.uk.

[b] Institute of Functional Nano & Soft Materials (FUNSOM), Joint International Research Laboratory of Carbon-Based Functional Materials and Devices, Soochow University, Suzhou, Jiangsu, 215123, P. R. China.

[c] Jiangsu Key Laboratory of Advanced Negative Carbon Technologies, Soochow University, Suzhou, Jiangsu, 215123, P. R. China.

[d] Department of Chemistry, Molecular Sciences Research Hub, Imperial College London, White City Campus, London, W12 0BZ, UK. E-mail: m.fuchter@imperial.ac.uk

[e] Dipartimento di Chimica e Chimica Industriale, Università di Pisa, 56124 Pisa, Italy

[f] Université Paris-Saclay, ENS Paris-Saclay, CNRS, PPSM, 91190, Gif-sur-Yvette, France.

[‡] These authors contributed equally to this work.

Table of Contents

General information..**S3**

Synthesis..**S8**

X-ray Crystallography **S33**

Photophysical characterization and computations...**S35**

Devices **S39**

Literature study...**S41**

References **S44**

General information:

General synthetic information.

All reagents and solvents for the synthesis and characterization were obtained from commercial sources. Anhydrous THF and toluene were obtained from an MBraun SPS5 solvent purification system. Other chemicals were used directly without additional purification. Air-sensitive reactions were conducted under a nitrogen atmosphere using Schlenk techniques. Flash column chromatography was carried out using silica gel (Silia-P from Silicycle, 60 Å, 40-63 μm). Analytical thin-layer-chromatography (TLC) was performed with silica plates with plastic backings (250 μm with F-254 indicator). TLC visualization was accomplished by 365 nm UV lamp. Melting points were measured using open-ended capillaries on an Electrothermal 1101D Mel-Temp apparatus and are uncorrected. HPLC analysis was conducted on a Shimadzu LC-40 HPLC system. HPLC traces were performed using a Shim-pack GIST 3μm C18 reverse phase analytical column. ^{1}H and ^{13}C NMR spectra were measured using Bruker AVII 400 or Bruker AVIII-HD 500 NMR spectrometers. The following abbreviations have been used for multiplicity assignments: "s" for singlet, "d" for doublet, "t" for triplet, "dd" for doublet of doublets and "m" for multiplet. Deuterated chloroform ($CDCl_3$), dichloromethane (CD_2Cl_2) or DMSO (DMSO-d_6) was used as the solvent of record. ^{1}H NMR and ^{13}C NMR spectra were referenced to the solvent peak. Electrospray ionization (ESI) and Matrix-assisted laser desorption/ionization (MALDI) high-resolution mass spectrometry (HRMS) spectra were obtained at the University of Edinburgh Mass Spectrometry Facility. Elemental analyses were performed at London Metropolitan University. TGA/DSC analyses were performed on a Netzsch STA449C instrument under nitrogen gas at a 10 K/min heating rate.

Theoretical Calculations.

All ground-state optimizations were carried out using Density Functional Theory (DFT) level with Gaussian 09[1] using the PBE0[2] functional and the 6-31G(d,p) basis set.[3] The simulated CD spectra, optimized geometry in S_1 states, electric and magnetic transition dipole moments were calculated using Time-Dependent DFT (TD-DFT) within the Tamm-Dancoff approximation

(TDA)[4, 5] at the M062X/6-31G(d,p) level of theory in THF. The transition state geometry was calculated at the M062X/6-31G(d,p) level. Frequency calculations identified this structure as a transition state as having only one imaginary frequency. Dissymmetry factor (*g*) for emission was calculated using equation (1):

$$g = \frac{4|\boldsymbol{\mu}||\boldsymbol{m}|}{|\boldsymbol{\mu}|^2+|\boldsymbol{m}|^2}\cos\theta \quad (1)$$

Where $\boldsymbol{\mu}$ and $\boldsymbol{m}$ refer to the electric transition dipole moment and magnetic transition dipole moment vectors, respectively. θ is the angle between $\boldsymbol{\mu}$ and $\boldsymbol{m}$.[6] Molecular orbitals were visualized using GaussView 6.0 software.[7] Vertical excited states were also calculated using Spin-Component Scaling second-order algebraic diagrammatic construction (SCS-ADC2)/cc-pVDZ calculations based on the ground-state optimized structure using DFT method.[8, 9] Difference density plots were used to visualize change in electronic density between the ground and excited state and were visualized using the VESTA package.[10] Calculations were submitted and processed using Silico V3,[11] which incorporates a number of publicly available software libraries, including: cclib[12] for parsing of result files, VMD[13]/Tachyon[14] for 3D rendering, Matplotlib[15] for drawing of graphs, Open Babel[16]/Pybel[17] for file interconversion and PySOC[18] for the calculation of spin-orbit coupling.

Electrochemical measurements.

Cyclic Voltammetry (CV) analysis was performed on an Electrochemical Analyzer potentiostat model 620E from CH Instruments at a sweep rate of 100 mV/s. Differential pulse voltammetry (DPV) was conducted with an increment potential of 0.01 V and a pulse amplitude, width, and period of 50 mV, 0.06, and 0.5 s, respectively. All measurements were performed in degassed DCM with 0.1 M tetra-n-butylammonium hexafluorophosphate ([^{n}Bu$_4$N]PF$_6$) as the supporting electrolyte and ferrocene/ferrocenium (Fc/Fc$^+$) as the internal reference (0.46 V vs SCE).[19] An Ag/Ag$^+$ electrode, a glassy carbon electrode and a platinum electrode were used as the reference electrode, working electrode and counter electrode, respectively. The HOMO and LUMO energies were determined using the relation the relation HOMO/LUMO = -(E_{ox}/ E_{red} vs Fc/Fc$^+$

+4.8),[20, 21] where E_{ox} and E_{red} are the oxidation and reduction peak potentials versus Fc/Fc^+, respectively, calculated from the DPV.

Photophysical measurements:

Optically dilute solutions of concentrations on the order of 10^{-5} M were prepared in HPLC grade solvent for absorption and emission analysis. Absorption spectra were recorded at room temperature on a Shimadzu UV-2600 double beam spectrophotometer. Molar absorptivity determination was verified by linear regression analysis of values obtained from four independent solutions at varying concentrations from 3×10^{-6} to 2×10^{-5} M. For emission studies, steady-state photoluminescence (PL) spectra and time-resolved PL decays in solution were recorded at 298 K using Edinburgh Instruments FS5 fluorophotometer. Degassed solutions were prepared via three freeze-pump-thaw cycles and spectra/decays were measured using a home-made Schlenk quartz cuvette. Samples were excited at 340 nm for steady-state measurements. Time-resolved PL measurements of solutions were carried out using the time-correlated single-photon counting (TCSPC) technique. The samples were excited at 375 nm with a pulsed laser diode. Photoluminescence quantum yields for solutions were determined using the optically dilute method,[22] in which four sample solutions with absorbances between 0.2 to 0.02 at 350 nm were used. The Beer-Lambert law was found to remain linear at the concentrations of the solutions. For each sample, linearity between absorption and emission intensity was verified through linear regression analysis with the Pearson regression factor (R^2) for the linear fit of the data set surpassing 0.9. Individual relative quantum yield values were calculated for each solution and the values reported represent the slope obtained from the linear fit of these results. The quantum yield of the sample, Φ_{PL}, can be determined by the equation $\Phi_{PL} = \left(\Phi_r * \frac{A_r}{A_s} * \frac{I_s}{I_r} * \frac{n_s^2}{n_r^2}\right)$, where A stands for the absorbance at the excitation wavelength (λ_{exc}: 350 nm), I is the integrated area under the corrected emission curve and n is the refractive index of the solvent with the subscripts “s” and “r” representing sample and reference respectively.[23] Φ_r is the absolute quantum yield of the external reference quinine sulfate (Φ_r = 54.6% in 0.5 M H_2SO_4).[24] The experimental uncertainty in the PL quantum yields is conservatively estimated to be 10%, though we have found that statistically we can reproduce

Φ_{PL} values to 3% relative error. The ΔE_{ST} of solution samples were estimated from the different in energy of the onsets of the SS PL and phosphorescence spectra at 77 K. Samples were excited by a xenon flashlamp emitting at 340 nm (EI FS5, SC-70). Phosphorescence spectra were measured with a time-gated window of 1-10 ms.

Thin doped films of emitters in a host matrix were spin-coated on a quartz substrate (for quantum yield measurements) or a sapphire substrate (for time-resolved PL measurements) using a spin speed of 1500 rpm for 60 s to give a thickness of ~80 nm. An integrating sphere (Edinburgh Instruments FS5, SC30 module) was employed for quantum yield measurements for thin film samples. The Φ_{PL} of the films were measured in air and then in N_2, by purging the integrating sphere with N_2 gas flow for 2 min. The photophysical properties of the film samples were measured using an Edinburgh Instruments FS5 fluorimeter. Time-resolved PL measurements of the thin films were carried out using the multi-channel scaling (MCS) and TCSPC technique. The samples were excited at 375 nm by a pulsed laser diode or a xenon flashlamp and were kept in a vacuum of $< 8 \times 10^{-4}$ mbar.

Enantiomers were isolated using a Chiralpak IE column (10 mm I.D. x 250 mm) with hexane:THF 95:5 as the mobile phase, a 5 ml min^{-1} flowrate and UV monitoring at 360 nm. Two successive injections were carried out to ensure the maximal purity in the sample. Subsequent analytical cHPLC revealed high enantiopurity (*ee.* 99% for (*P*)-**tBuPh-BN**, 97% for (*M*)-**tBuPh-BN**, 99% for (*P*)-**DPA-tBuPh-BN** and 99% for (*M*)-**DPA-tBuPh-BN**) (Figures **S24** and **S25**). Circular dichroism (CD) measurements were conducted using an Applied Photophysics Chirascan spectrophotometer in 4.7×10^{-6} M toluene solutions at a measuring rate of 1 s per point. The dissymmetry factor (g_{abs}) was calculated using the equation $g_{abs} = \Delta A/A$, where $A = 0.5 \times (A_L + A_R)$ and represents the UV-Vis absorption of the sample. A_L and A_R represent the absorption of left- and right-handed light, respectively. ΔA is calculated through the following equation ΔA = CD (mdeg) / 32980, where CD (mdeg) represents the CD signal in mdeg units. Circularly polarized luminescence (CPL) measurements were performed using a homemade spectrofluoropolarimeter.[25] The spectra were run in 1×10^{-5} M solutions in THF. The samples were excited by 365 nm radiation (LED source) with a 90° geometry

between excitation and detection. The exciting beam was linearly polarized parallel to the direction of detection. The spectra were baseline-corrected by subtraction of the spectrum of the racemic compound. Parameters used: bandwidth 6 nm, scan speed 1 nm/sec, integration time 2 sec, PMT voltage 500 V, accumulations 6.

Fitting of the time-resolved PL decays:

Time-resolved PL measurements were fitted to a sum of exponentials decay model, with chi-squared (χ^2) values between 1 and 2, using the EI FS5 software. Each component of the decay is assigned a weight, (w_i), which is the contribution of the emission from each component to the total emission.

The average lifetime was then calculated using the following:

- Two exponential decay model:

$$\tau_{AVG} = \tau_1 w_1 + \tau_2 w_2$$

with weights defined as $w_1 = \frac{A1\tau_1}{A1\tau_1 + A2\tau_2}$ and $w_2 = \frac{A2\tau_2}{A1\tau_1 + A2\tau_2}$ where $A1$ and $A2$ are the preexponential-factors of each component.

- Three exponential decay model:

$$\tau_{AVG} = \tau_1 w_1 + \tau_2 w_2 + \tau_3 w_3$$

with weights defined as $w_1 = \frac{A1\tau_1}{A1\tau_1 + A2\tau_2 + A3\tau_3}$, $w_2 = \frac{A2\tau_2}{A1\tau_1 + A2\tau_2 + A3\tau_3}$ and $w_3 = \frac{A3\tau_3}{A1\tau_1 + A2\tau_2 + A3\tau_3}$ where $A1$, $A2$ and $A3$ are the preexponential-factors of each component.

OLED Fabrication and Characterization:

OLEDs were fabricated on the indium-tin oxide (ITO) coated transparent glass substrates with multiple layers. The ITO glass substrates have a thickness of ca. 100 nm and a sheet resistance of ca. 30 Ω per square and were cleaned with optical detergent, deionized water, acetone, and isopropanol successively and then dried in an oven. For vacuum-evaporated OLEDs, the ITO

substrates were exposed to UV ozone for 15 minutes initially. All the organic materials were thermally evaporated at a rate of 1 Å s^{-1} under a vacuum of ca. 10^{-5} Torr. Finally, LiF and Al were successively deposited at a rate of 0.1 Å s^{-1} and 5 Å s^{-1}, respectively. Four identical OLED devices were formed on each of the substrates and the emission area of 0.09 cm^2 for each device. The EL performances of the devices were measured with a PHOTO RESEARCH Spectra Scan PR 655 PHOTOMETER and a KEITHLEY 2400 Source Meter constant current source at room temperature.

Synthesis:

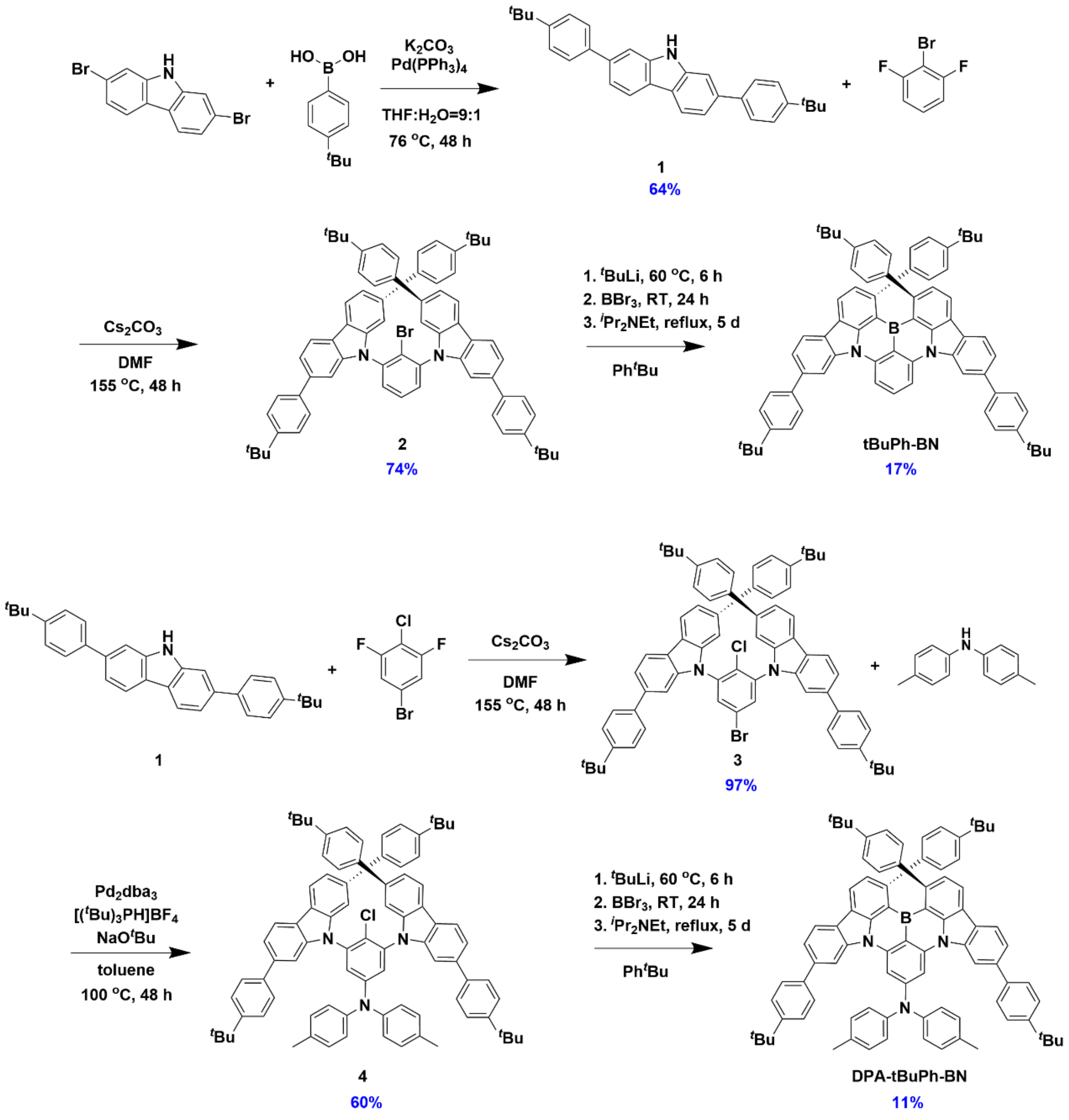

Figure **S1**. Synthesis scheme of **tBuPh-BN** and **DPA-tBuPh-BN**.

2,7-bis(4-(*tert*-butyl)phenyl)-9*H*-carbazole (1)

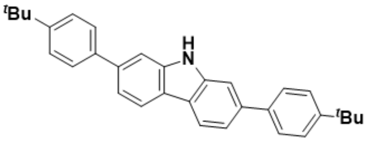

2,7-dibromo-9*H*-carbazole (2.00 g, 6.15 mmol, 1 equiv.), (4-*tert*-butyl)phenyl) boronic acid (2.41 g, 13.5 mmol, 2.2 equiv.) and $Pd(PPh_3)_4$ (356 mg, 308 μmol, 0.05 equiv.) were added to a mixture of THF (36 mL) and 5 mol L^{-1} potassium carbonate aqueous solution (4 mL). The reaction was stirred at 76 °C under nitrogen for 48 hours. After cooling to room temperature, the reaction was quenched by adding water and extracted with 3× 50 mL dichloromethane. The combined organic phase was then concentrated under reduced pressure. The crude product was washed with hexane and methanol to obtain compound **1** as a beige powder. **Yield** 64%, 1.70 g. **Mp**: 324-325 °C. **R_f**: 0.1 (dichloromethane: hexane = 1:4). **^{1}H NMR (400 MHz, DMSO-*d*^{6})** δ 11.37 (s, 1H), 8.17 (d, J = 8.1 Hz, 2H), 7.73 – 7.65 (m, 6H), 7.55 – 7.49 (m, 4H), 7.46 (dd, J = 8.2, 1.6 Hz, 2H), 1.34 (s, 18H). **^{13}C NMR (126 MHz, DMSO-*d*^{6})** δ 149.97, 141.40, 138.79, 138.25, 127.12, 126.19, 121.93, 121.08, 118.37, 109.11, 34.72, 31.64. **HRMS (ESI-MS)**: $[C_{32}H_{33}N + H]^+$ **Calculated**: 432.2686; **Found:** 432.2684.

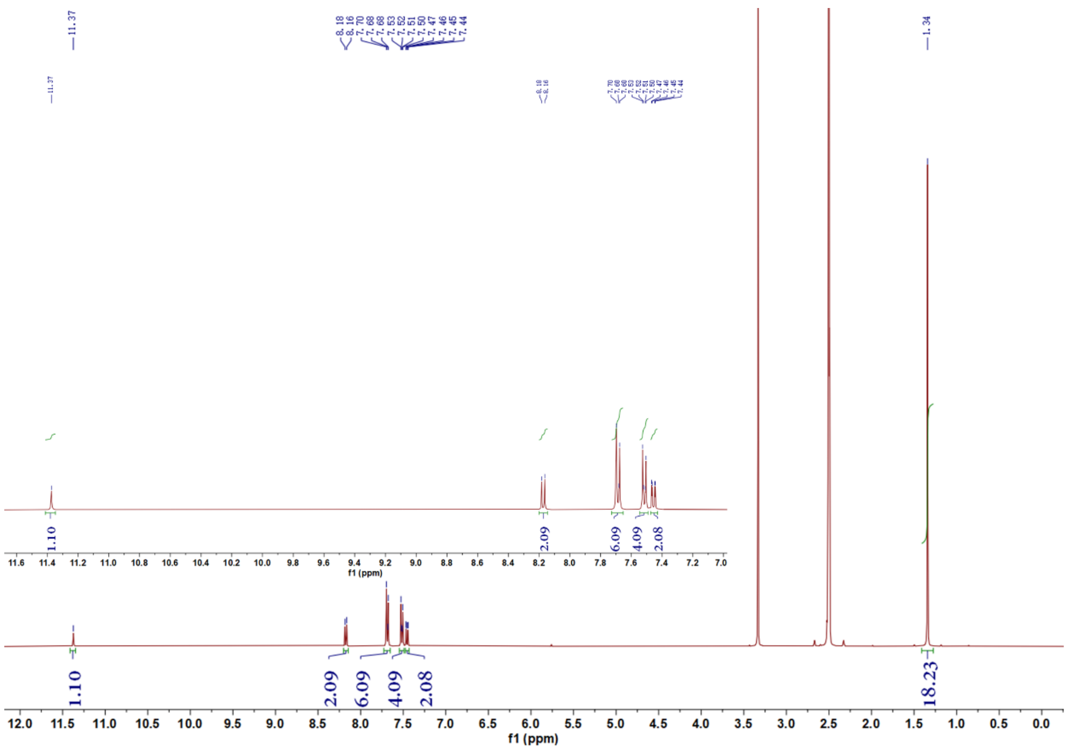

Figure **S2**. ^{1}H NMR spectrum of **1** in DMSO-d^6.

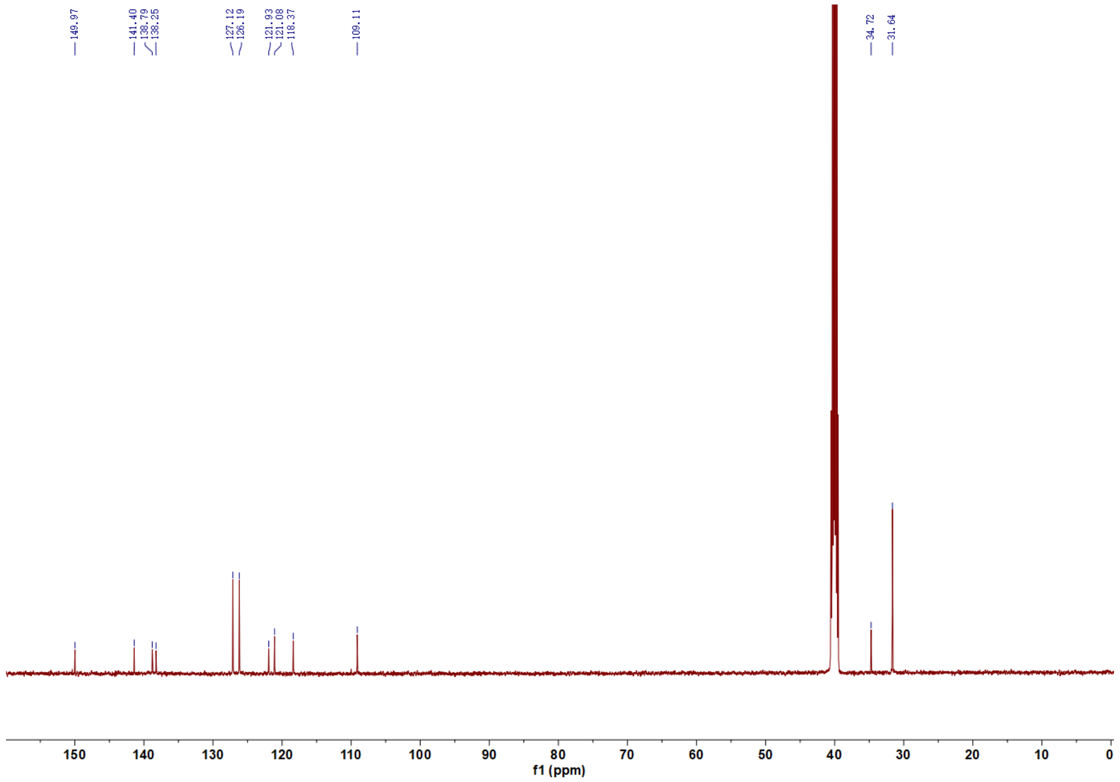

Figure **S3**. ^{13}C NMR spectrum of **1** in DMSO-d^6.

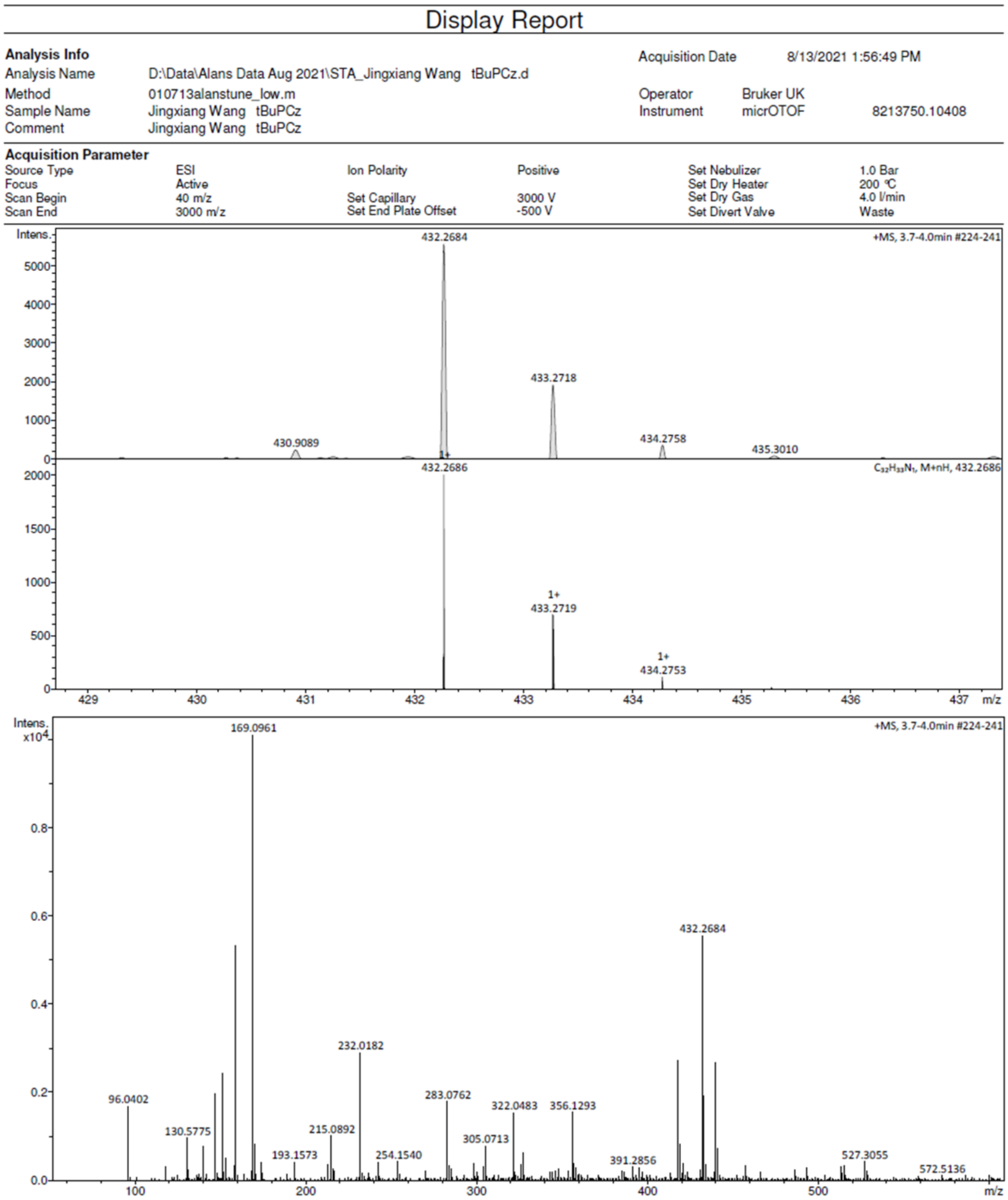

Figure **S4**. HRMS spectrum of **1**.

9,9'-(2-bromo-1,3-phenylene)bis(2,7-bis(4-(*tert*-butyl)phenyl)-9*H*-carbazole) (2)

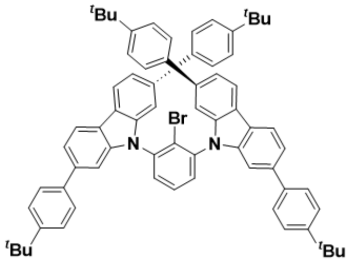

Compound **1** (492 mg, 1.14 mmol, 2.2 equiv.), 2-bromo-1,3-difluorobenzene (100 mg, 0.518 mmol, 1 equiv.), cesium carbonate (844 mg, 2.59 μmol, 5 equiv.) and DMF (8 mL) were added to a 2-neck Schlenk tube. The mixture was stirred at 155 °C under nitrogen for 48 hours. After cooling to room temperature, the reaction mixture reaction was quenched by adding water and extracted with 3× 50 mL dichloromethane. The combined organic phase was then concentrated under reduced pressure. The crude product was washed with methanol and hexane to obtain compound **2** as a white solid. **Yield** 74%, 389 mg. **Mp**: > 395 °C. **R*f***: 0.4 (dichloromethane: hexane= 1: 4). **^{1}H NMR (400 MHz, $CDCl_3$)** δ 8.24 – 8.19 (m, 4H), 7.74 (s, 3H), 7.59 (dd, J = 8.3, 2.7 Hz, 12H), 7.43 (d, J = 8.5 Hz, 8H), 7.38 (d, J = 1.0 Hz, 4H), 1.33 (s, 36H). **^{13}C NMR (126 MHz, $CDCl_3$)** δ 150.16, 141.78, 139.57, 139.30, 138.91, 131.35, 129.62, 127.17, 125.75, 125.61, 122.40, 120.66, 120.12, 108.48, 34.53, 31.40. **HRMS (ESI-MS)**: $[C_{70}H_{67}BrN_2 + NH_4]^+$ **Calculated**: 1032.4826; **Found:** 1032.4782.

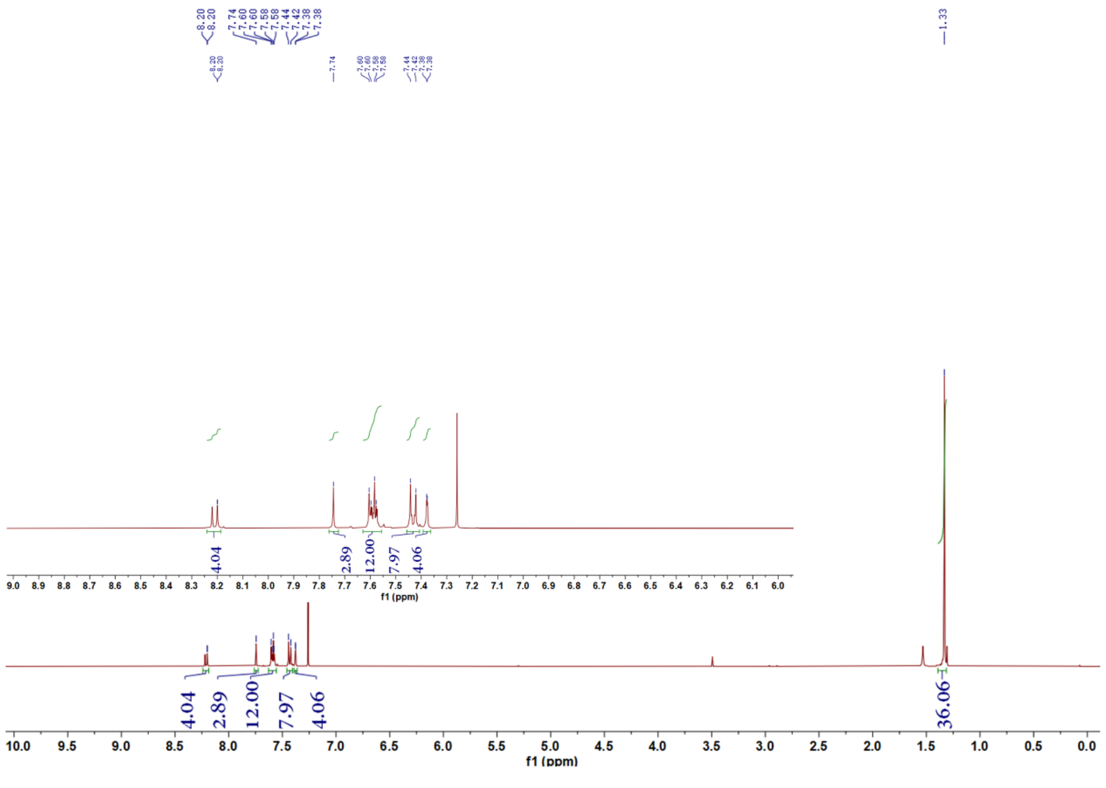

Figure **S5**. ^{1}H NMR spectrum of **2** in $CDCl_3$.

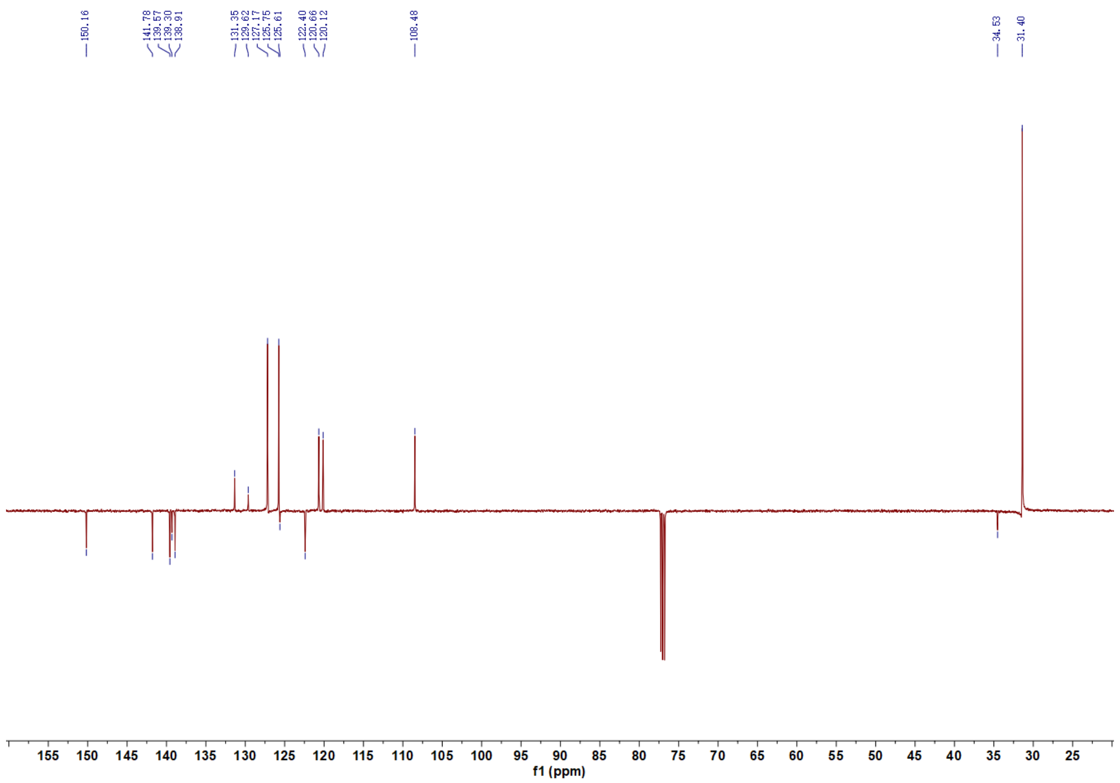

Figure **S6**. DEPTQ ^{13}C NMR spectrum of **2** in $CDCl_3$.

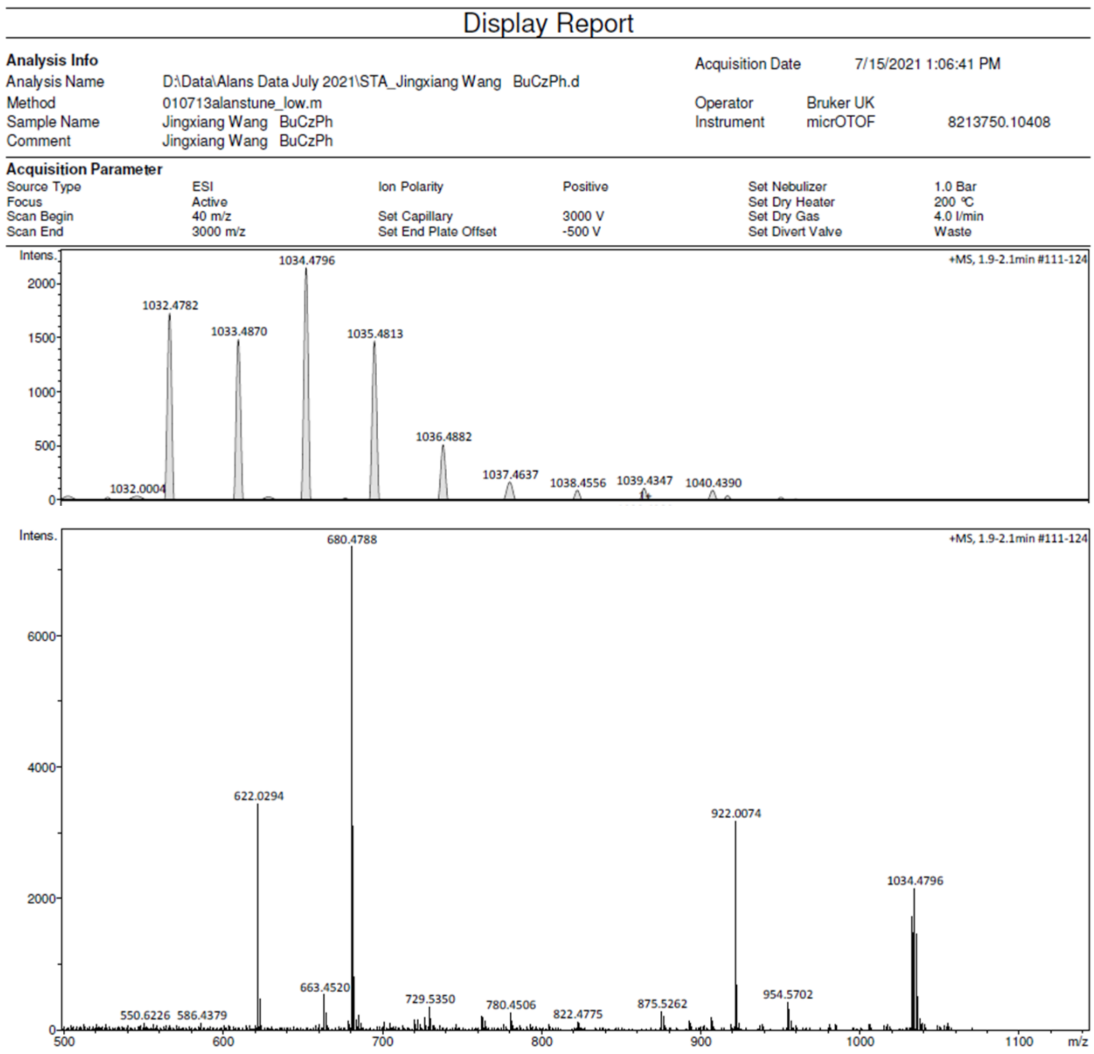

Figure **S7**. HRMS spectrum of **2**.

tBuPh-BN

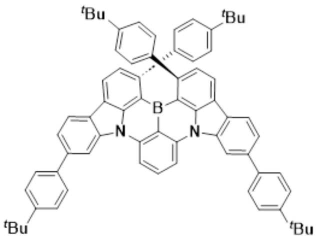

A solution of *t*-butyllithium in pentane (0.868 mL, 1.7 M, 1.48 mmol, 3 equiv.) was added slowly to a solution of **2** (0.500 g, 0.492 mmol, 1.0 equiv.) in *tert*-butylbenzene (11 mL) at -

78 °C under a nitrogen atmosphere. Then the reaction was stirred at 60 °C for 6 h. After that, boron tribromide (0.698 mL, 7.38 mmol, 15 equiv.) was added slowly at -78 °C. The reaction mixture was then allowed to warm to room temperature and stirred for 24 h. *N*,*N*-diisopropylethylamine (0.514 mL, 2.95 mmol, 6 equiv.) was then added at 0 °C. After stirring under reflux for 5 days, the reaction mixture was quenched by adding 50 mL water and extracted with 3× 50 mL dichloromethane. The organic phase was then separated and concentrated under reduced pressure. The crude product was purified by column chromatography on silica gel (dichloromethane: hexane = 1: 8) to afford **tBuPh-BN** as a yellow powder. **Yield** 17%, 79 mg. **Mp**: 340-342 °C. **R_f**: 0.3 (dichloromethane: hexane = 1: 5). **^{1}H NMR (500 MHz, $CDCl_3$)** δ 8.68 (s, 2H), 8.38 (d, *J* = 8.3 Hz, 2H), 8.28 (d, *J* = 8.0 Hz, 2H), 8.15 (d, *J* = 7.8 Hz, 2H), 8.04 (t, *J* = 8.2 Hz, 1H), 7.79 (d, *J* = 8.4 Hz, 4H), 7.71 (dd, *J* = 8.0, 1.1 Hz, 2H), 7.61 (d, *J* = 8.4 Hz, 4H), 7.32 (d, *J* = 7.8 Hz, 2H), 6.87 (d, *J* = 7.5 Hz, 4H), 6.70 (d, *J* = 8.4 Hz, 4H), 1.44 (s, 18H), 1.29 (s, 18H). **^{13}C NMR (126 MHz, $CDCl_3$)** δ 150.51, 149.90, 146.95, 143.01, 141.26, 140.40, 139.95, 139.19, 137.34, 132.46, 131.39, 127.41, 126.04, 125.95, 123.98, 123.16, 122.38, 121.71, 121.54, 121.06, 120.12, 113.13, 108.19, 34.66, 34.35, 31.46, 31.32. **HRMS (ESI-MS)**: $[C_{70}H_{65}BN_2 + H]^+$ **Calculated**: 945.5314; **Found:** 945.5313. **Anal. Calcd. For $C_{70}H_{65}BN_2$**: C 88.96%, H 6.93%, N 2.96% **Found**: C 89.12%, H 6.24%, N 2.43%. **HPLC** (80% Tetrahydrofuran and 20% Water): 99.29% pure, retention time 10.391 min.

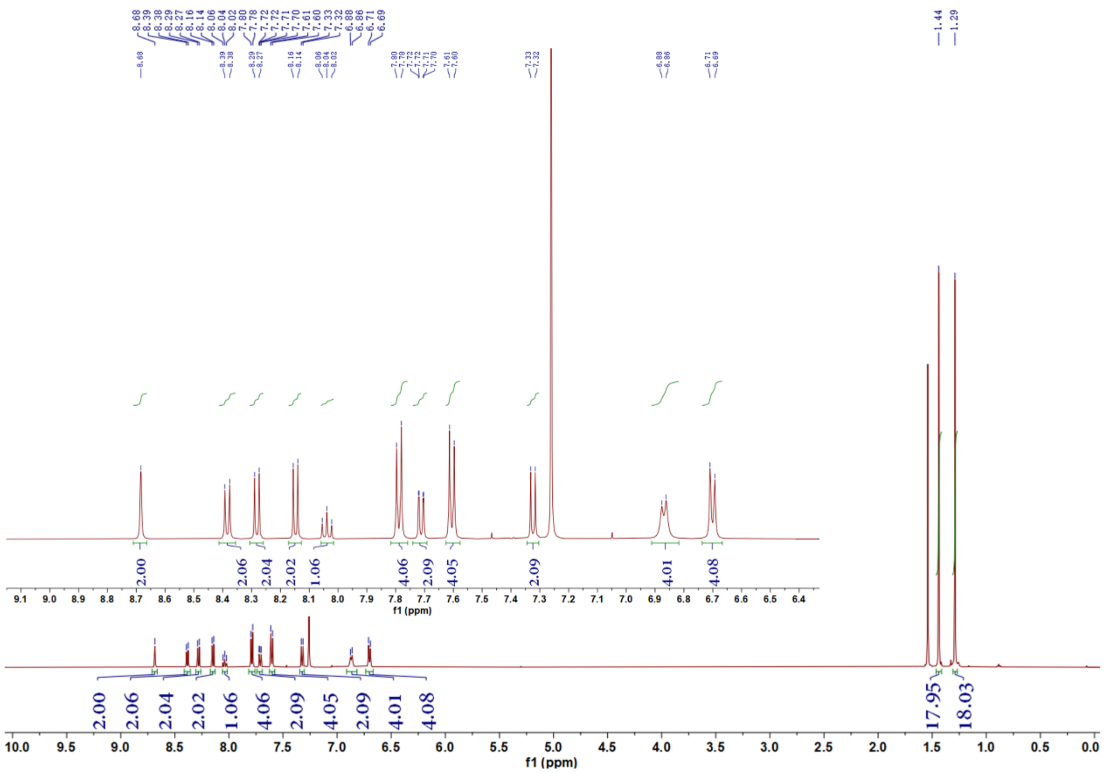

Figure **S8**. ^{1}H NMR spectrum of **tBuPh-BN** in $CDCl_3$.

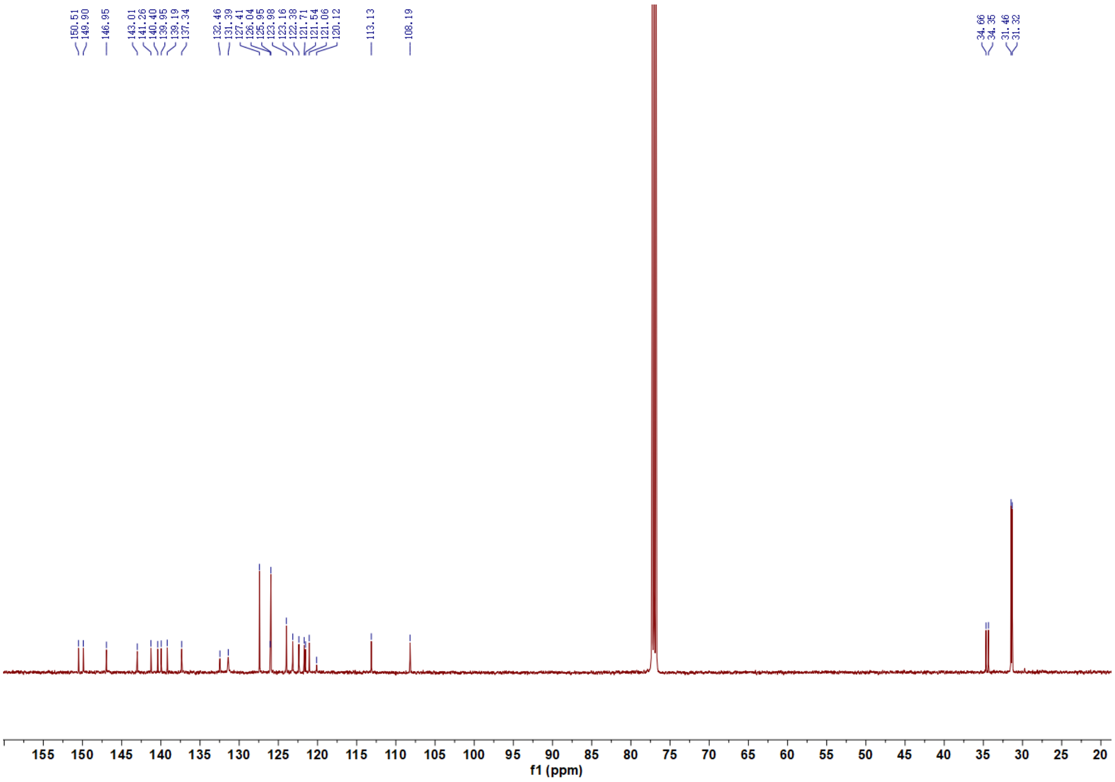

Figure **S9**. ^{13}C NMR spectrum of **tBuPh-BN** in $CDCl_3$.

Display Report

Analysis Info

Analysis Name	D:\Data\Alans Data Sept 2021\STA_Jingxiang Wang tBuPhBN.d	Acquisition Date	9/22/2021 3:05:10 PM
Method	010713alanstune_low.m	Operator	Bruker UK
Sample Name	Jingxiang Wang tBuPhBN	Instrument	micrOTOF 8213750.10408
Comment	Jingxiang Wang tBuPhBN		

Acquisition Parameter

Source Type	ESI	Ion Polarity	Positive	Set Nebulizer	1.0 Bar
Focus	Active			Set Dry Heater	200 °C
Scan Begin	40 m/z	Set Capillary	3000 V	Set Dry Gas	4.0 l/min
Scan End	3000 m/z	Set End Plate Offset	-500 V	Set Divert Valve	Waste

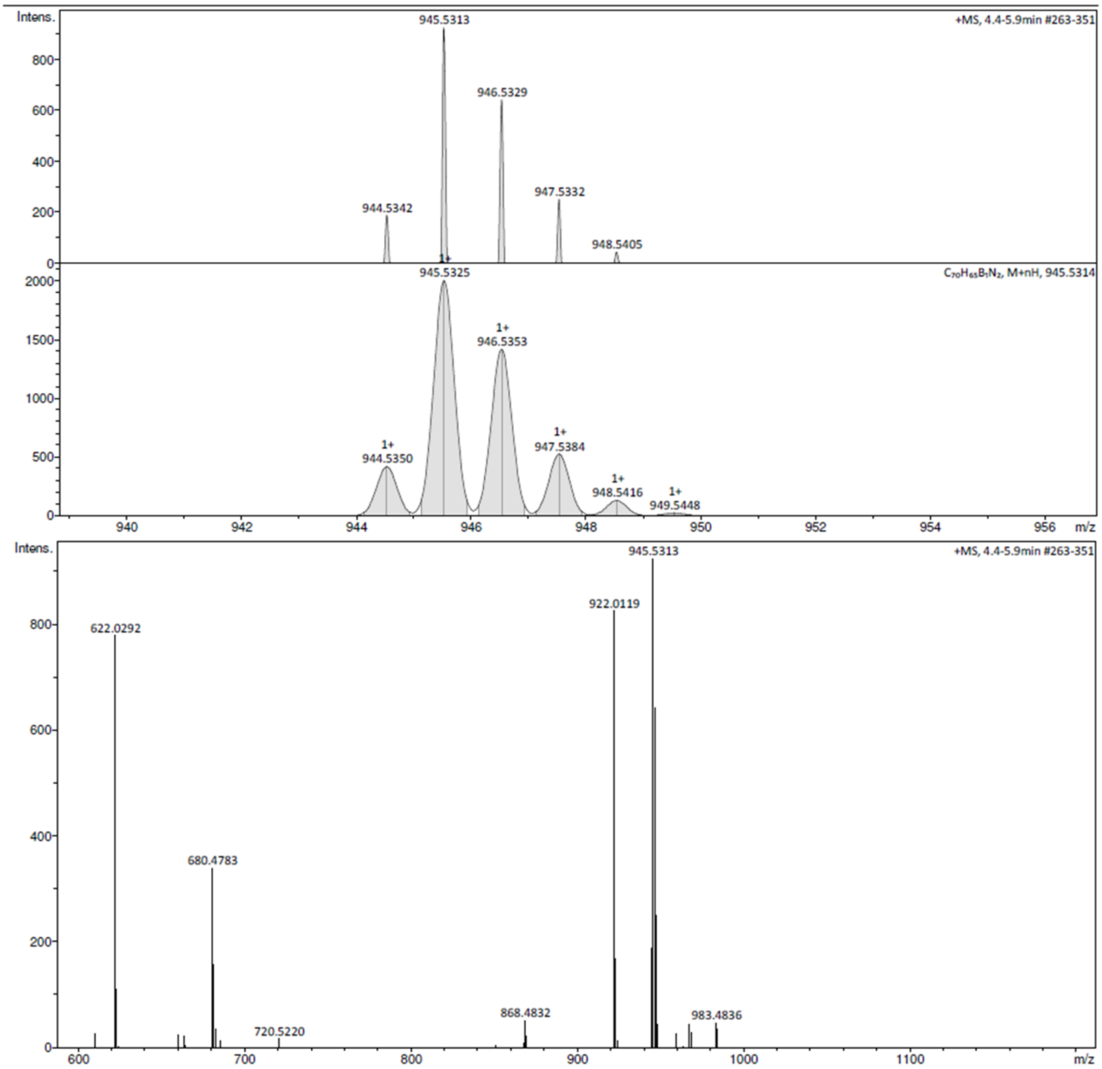

Figure **S10**. HRMS spectrum of **tBuPh-BN**.

LONDON METROPOLITAN UNIVERSITY
HUMAN SCIENCES

Elemental Analysis Sample Results

**Name**	Jingxiang Wang
**Organisation Name**	University of St Andrews
**Purchase order number**	

**Standard – Acetanilide**		
**Element**	**Expected %**	**Found**
**Carbon**	71.10 (+/- 0.23)	71.02
**Hydrogen**	6.71 (+/- 0.07)	6.64
**Nitrogen**	10.34 (+/- 0.09)	10.29

**Analysis – tBuPh-BN**			
**Element**	**Expected %**	**Found (1)**	**Found (2)**
**Carbon**	88.96	89.08	89.17
**Hydrogen**	6.93	6.39	6.09
**Nitrogen**	2.96	2.48	2.37

**Date completed**	16.04.2024
**Signature**	O. McCullough
**Comments**	

Figure **S11**. Elemental analysis data of **tBuPh-BN**.

HPLC Trace Report02Aug2023

<Sample Information>

Sample Name	: tbuphbn		
Sample ID	:		
Method Filename	: 80% THF 20% water 0.6 mlmin 20 mins.lcm		
Batch Filename	: 01082023.lcb		
Vial #	: 2-8	Sample Type	: Unknown
Injection Volume	: 5 uL		
Date Acquired	: 02/08/2023 13:35:36	Acquired by	: System Administrator
Date Processed	: 02/08/2023 13:55:39	Processed by	: System Administrator

<Chromatogram>

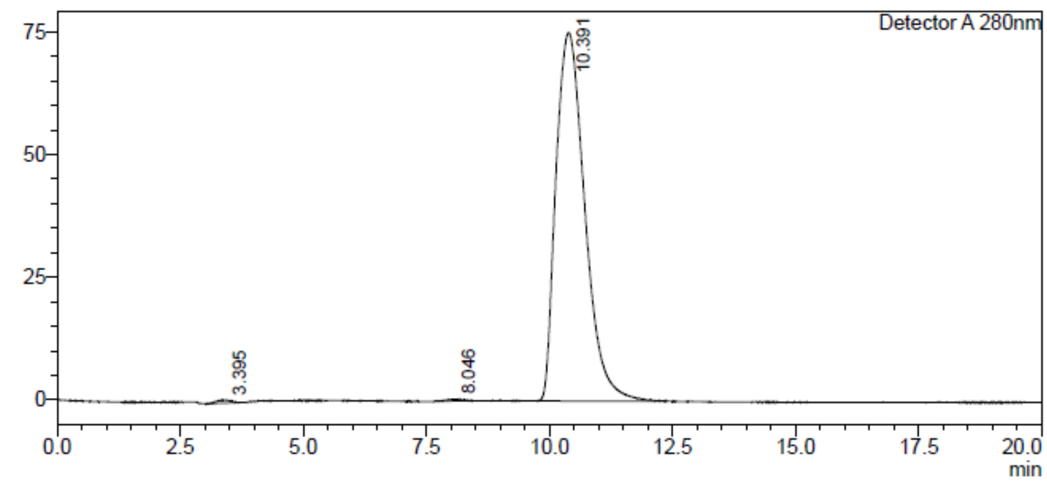

<Peak Table>

Detector A 280nm

Peak#	Ret. Time	Area	Height	Area%	Area/Height	Width at 5% Height
1	3.395	13253	616	0.430	21.531	0.589
2	8.046	8751	355	0.284	24.669	0.683
3	10.391	3061798	75061	99.286	40.791	1.330
Total		3083802	76031	100.000		

Figure **S12**. HPLC spectrum of **tBuPh-BN**.

9,9'-(5-bromo-2-chloro-1,3-phenylene)bis(2,7-bis(4-(*tert*-butyl)phenyl)-9*H*-carbazole) (3)

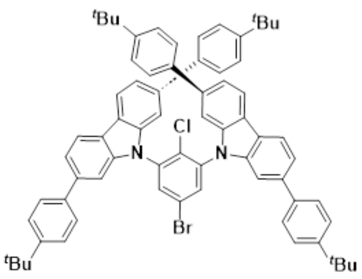

Compound **1** (1.59 g, 3.69 mmol, 2.1 equiv.), 5-bromo-2-chloro-1,3-difluorobenzene (400 mg, 1.76 mmol, 1 equiv.), cesium carbonate (2.87g, 8.79 mmol, 5 equiv.) and DMF (15 mL) were added to a 2-neck Schlenk tube. The mixture was stirred at 155 °C under nitrogen for 72 hours. After cooling to room temperature, the reaction mixture reaction was quenched by adding water and extracted with 3× 50 mL dichloromethane. The organic phase was then separated and concentrated under reduced pressure. The crude product was washed with methanol and hexane to obtain compound **3** as a pale yellow solid. **Yield** 97%, 1.80 g. **Mp**: 382-384 °C. **R*f***: 0.5 (dichloromethane: hexane= 1: 3.5). **^{1}H NMR (400 MHz, $CDCl_3$)** δ 8.20 (d, *J* = 8.0 Hz, 4H), 7.93 (s, 2H), 7.60 (d, *J* = 8.5 Hz, 12H), 7.44 (d, *J* = 8.5 Hz, 8H), 7.40 – 7.36 (m, 4H), 1.34 (s, 36H). **^{13}C NMR (126 MHz, $CDCl_3$)** δ 150.29, 141.61, 139.81, 138.75, 138.46, 134.05, 133.00, 127.18, 125.80, 122.60, 120.75, 120.49, 108.27, 34.55, 31.40. **HRMS (ESI-MS)**: $[C_{70}H_{66}BrClN_2 + H]^+$ **Calculated**: 1049.4171; **Found:** 1049.4154. $[C_{70}H_{66}{}^{81}BrClN_2 + NH_4]^+$ **Calculated**: 1068.4416; **Found:** 1068.4431.

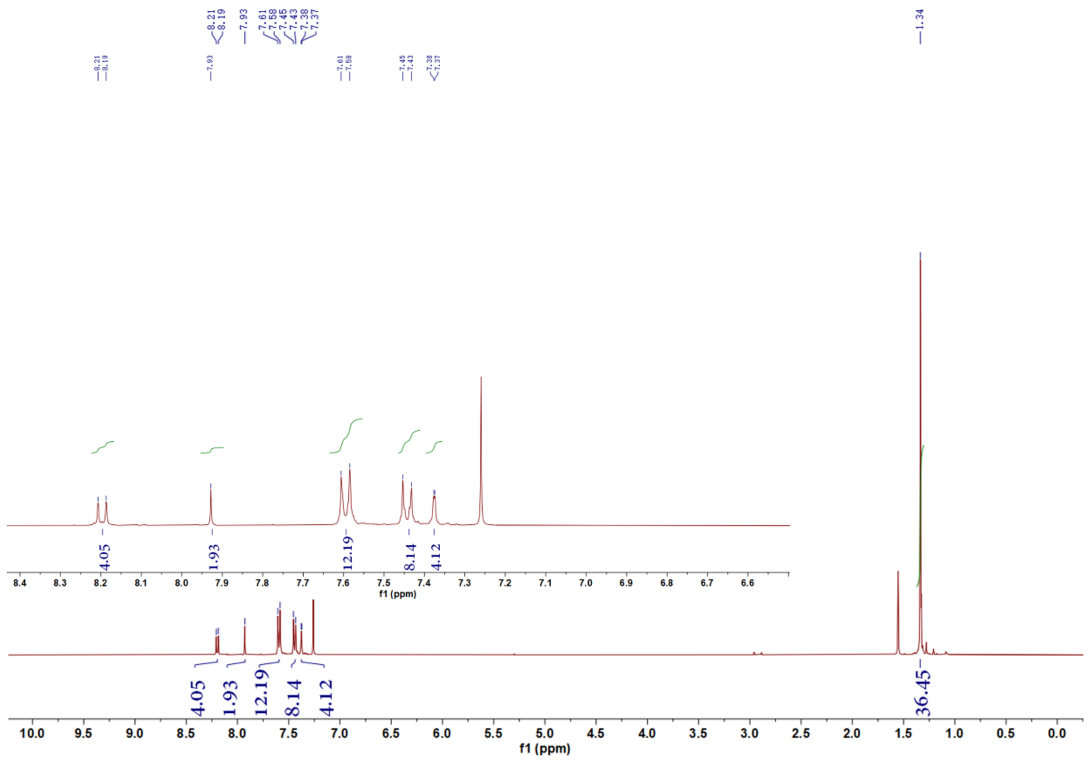

Figure **S13**. ^{1}H NMR spectrum of **3** in $CDCl_3$.

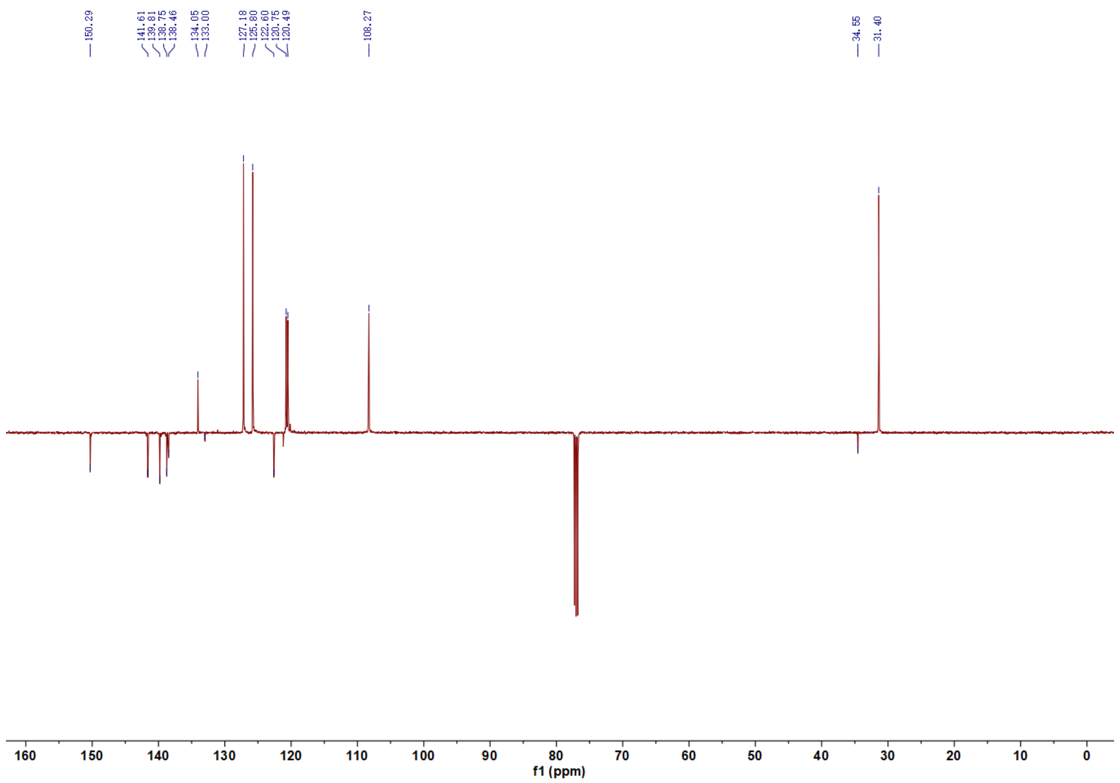

Figure **S14**. DEPTQ ^{13}C NMR spectrum of **3** in $CDCl_3$.

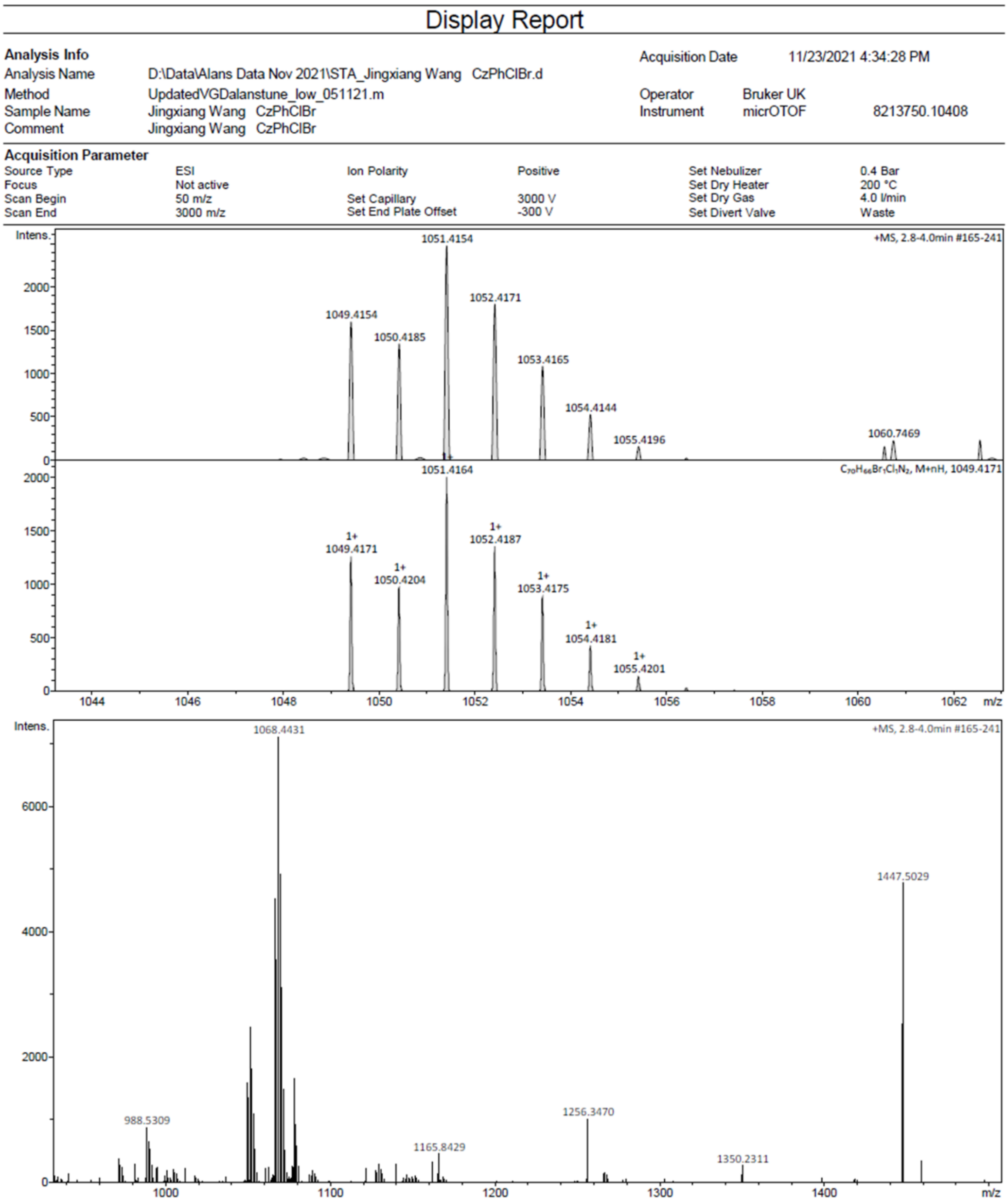

Figure **S15**. HRMS spectrum of **3**.

3,5-bis(2,7-bis(4-(*tert*-butyl)phenyl)-9*H*-carbazol-9-yl)-4-chloro-*N*,*N*-di-*p*-tolylaniline (4)

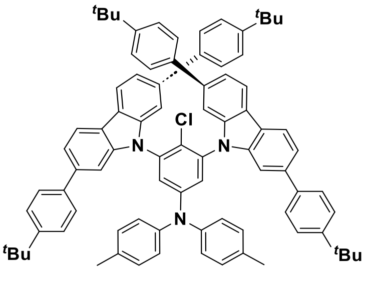

Compound **3** (700 mg, 0.666 mmol, 1.0 equiv.), di-*p*-tolylamine (145 mg, 0.733 mmol, 1.1 equiv.), $Pd_2(dba)_3$ (18.3 mg, 0.020 mmol, 0.03 equiv.), tri-*tert*-butylphosphonium tetrafluoroborate (9.83 mg, 0.0533 mmol, 0.08 equiv.), NaO^{t}Bu (192 mg, 2.00 mmol, 3 equiv.) and toluene (20 mL) were added to a 2-neck Schlenk tube. The mixture was stirred at 105 °C for 48 h under a nitrogen atmosphere. After cooling to room temperature, the reaction mixture reaction was quenched by adding water and extracted with 3× 50 mL dichloromethane. The organic phase was then separated and concentrated under reduced pressure. The crude product was purified by column chromatography on silica gel (dichloromethane: hexane= 1: 5) to afford compound **4** as a white solid. **Yield** 60%, 470 mg. **Mp**: decomposes at 364 °C. **R*f***: 0.4 (dichloromethane: hexane= 1: 2). **^{1}H NMR (500 MHz, CD_2Cl_2)** δ 8.17 (d, *J* = 8.1 Hz, 4H), 7.62 (d, *J* = 8.3 Hz, 8H), 7.55 (dd, *J* = 8.1, 1.2 Hz, 4H), 7.51 – 7.45 (m, 12H), 7.29 (s, 2H), 7.12 (d, *J* = 8.3 Hz, 4H), 6.86 (d, *J* = 8.2 Hz, 4H), 2.13 (s, 6H), 1.36 (s, 36H). **^{13}C NMR (126 MHz, CD_2Cl_2)** δ 150.32, 148.88, 143.61, 141.68, 139.40, 138.84, 137.12, 134.71, 130.33, 127.06, 125.78, 125.57, 122.23, 121.54, 121.33, 120.51, 119.84, 108.76, 34.44, 31.15, 20.52. **HRMS (ESI-MS)**: $[C_{84}H_{80}ClN_3 +H]^+$ **Calculated**: 1166.6114; **Found:** 1166.6080.

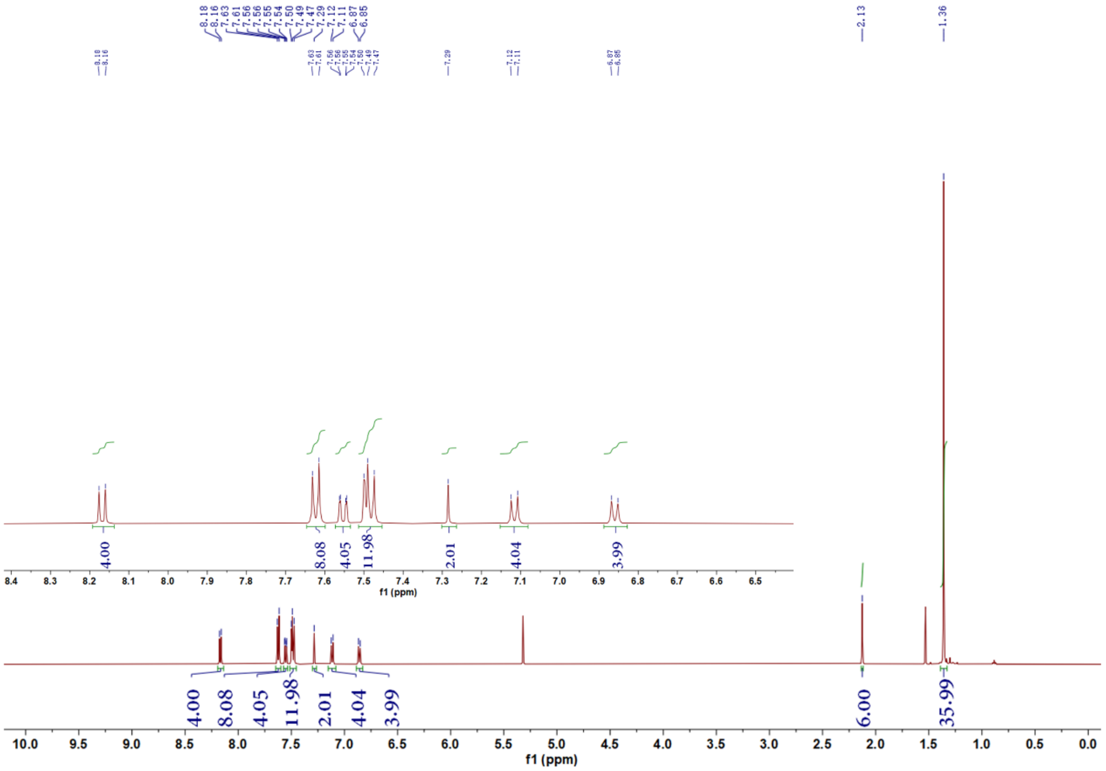

Figure **S16**. ^{1}H NMR spectrum of **4** in CD_2Cl_2.

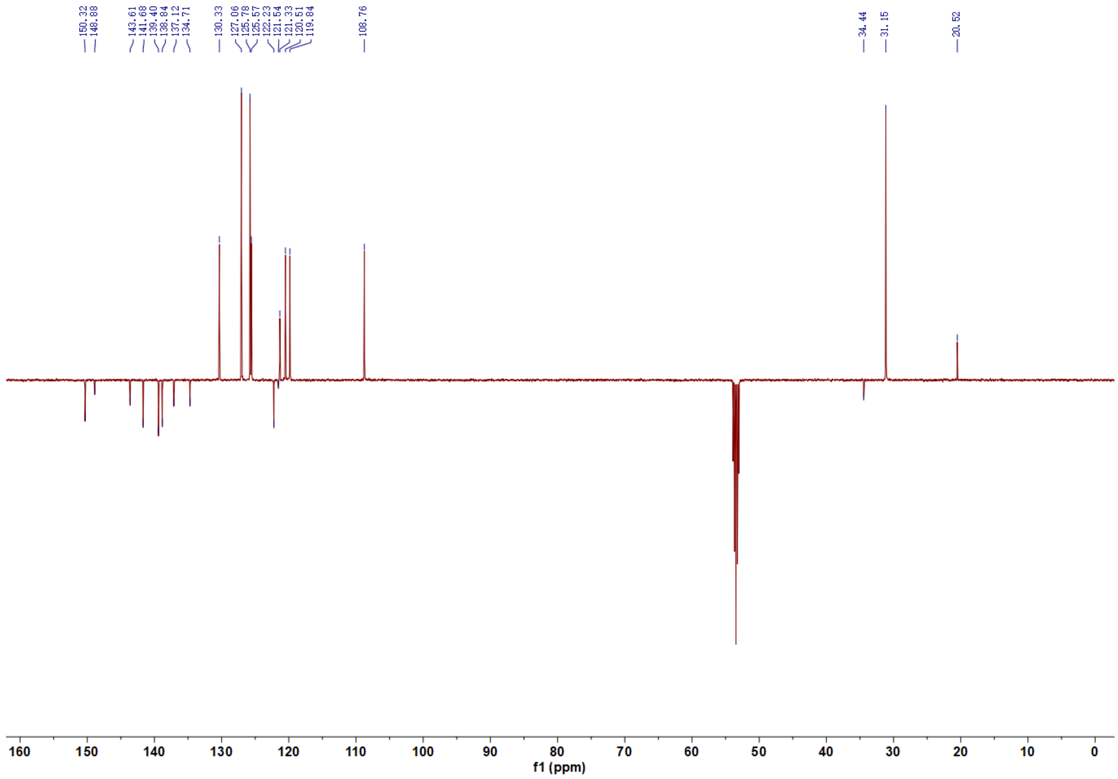

Figure **S17**. DEPTQ ^{13}C NMR spectrum of **4** in CD_2Cl_2.

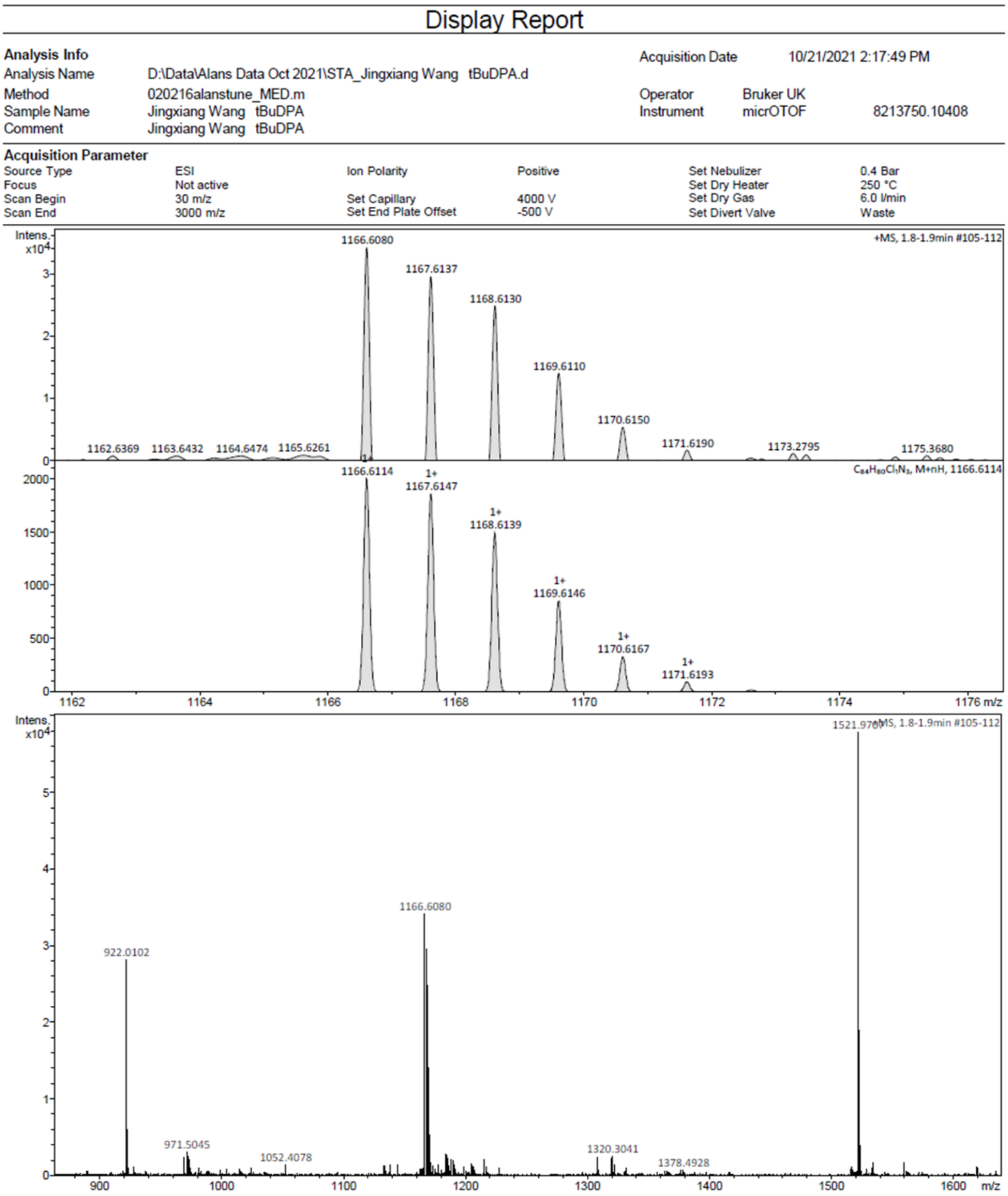

Figure **S18**. HRMS spectrum of **4**.

DPA-tBu-PhBN

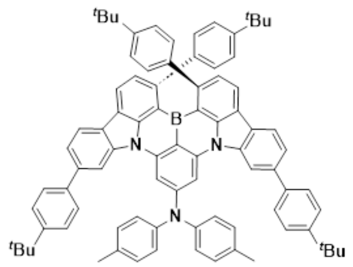

A solution of *t*-butyllithium in pentane (0.756 mL, 1.7 M, 1.29 mmol, 3 equiv.) was added slowly to a solution of **4** (0.500 g, 0.428 mmol, 1.0 equiv.) in *tert*-butylbenzene (12 mL) at -78 °C under a nitrogen atmosphere. Then the reaction was stirred at 60 °C for 6 h. After that, boron tribromide (0.527 mL, 5.57 mmol, 13 equiv.) was added slowly at -78 °C. The reaction mixture was then allowed to warm to room temperature and stirred for 24 h. *N*,*N*-diisopropylethylamine (0.448 mL, 2.57 mmol, 6 equiv.) was then added at 0 °C. After stirring under reflux for 5 days, the reaction mixture was quenched by adding water and extracted with 3× 50 mL dichloromethane. The organic phase was then separated and concentrated under reduced pressure. The crude product was purified by column chromatography on silica gel (chloroform: hexane = 1: 6) and washed with DCM to afford **DPA-tBuPh-BN** as a yellow powder. **Yield** 11%, 52 mg. **Mp**: 343-344 °C. **R*f***: 0.4 (chloroform: hexane = 1: 2). **^{1}H NMR (500 MHz, CD_2Cl_2)** δ 8.22 (d, *J* = 8.0 Hz, 2H), 8.17 – 8.08 (m, 4H), 7.99 (s, 2H), 7.62 (d, *J* = 7.9 Hz, 2H), 7.53 (d, *J* = 8.1 Hz, 4H), 7.42 (d, *J* = 8.1 Hz, 4H), 7.36 (d, *J* = 8.0 Hz, 4H), 7.29 (d, *J* = 7.8 Hz, 2H), 7.22 (d, *J* = 7.9 Hz, 4H), 6.86 (d, *J* = 6.2 Hz, 4H), 6.69 (d, *J* = 8.0 Hz, 4H), 2.25 (s, 6H), 1.44 (s, 18H), 1.28 (s, 18H). **^{13}C NMR (126 MHz, CD_2Cl_2)** δ 152.70, 150.29, 149.86, 146.69, 144.36, 143.47, 142.27, 140.22, 139.44, 138.49, 137.49, 134.75, 131.21, 130.42, 127.01, 126.57, 125.99, 125.86, 123.94, 123.05, 121.84, 121.48, 121.25, 120.86, 120.64, 112.25, 99.73, 34.50, 34.18, 31.20, 31.03, 21.04. **HRMS (MALDI-MS)**: $[C_{84}H_{78}BN_3]^+$ **Calculated**:1139.6283; **Found:** 1139.6252. **Anal. Calcd. For $C_{84}H_{78}BN_3$**: C 88.47%, H 6.89%, N 3.68% **Found**: C 88.36%, H 6.87%, N 3.43%. **HPLC** (80% Tetrahydrofuran and 20% Water): 97.98% pure, retention time 11.039 min.

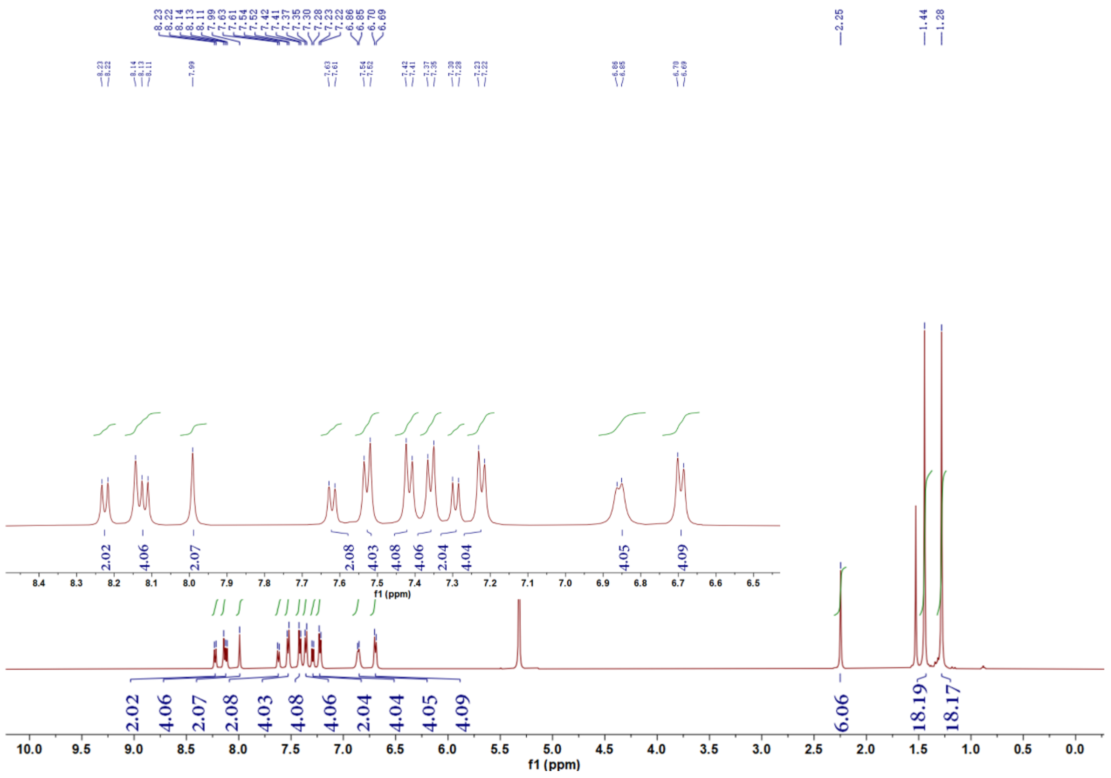

Figure **S19**. ^{1}H NMR spectrum of **DPA-tBuPh-BN** in CD_2Cl_2.

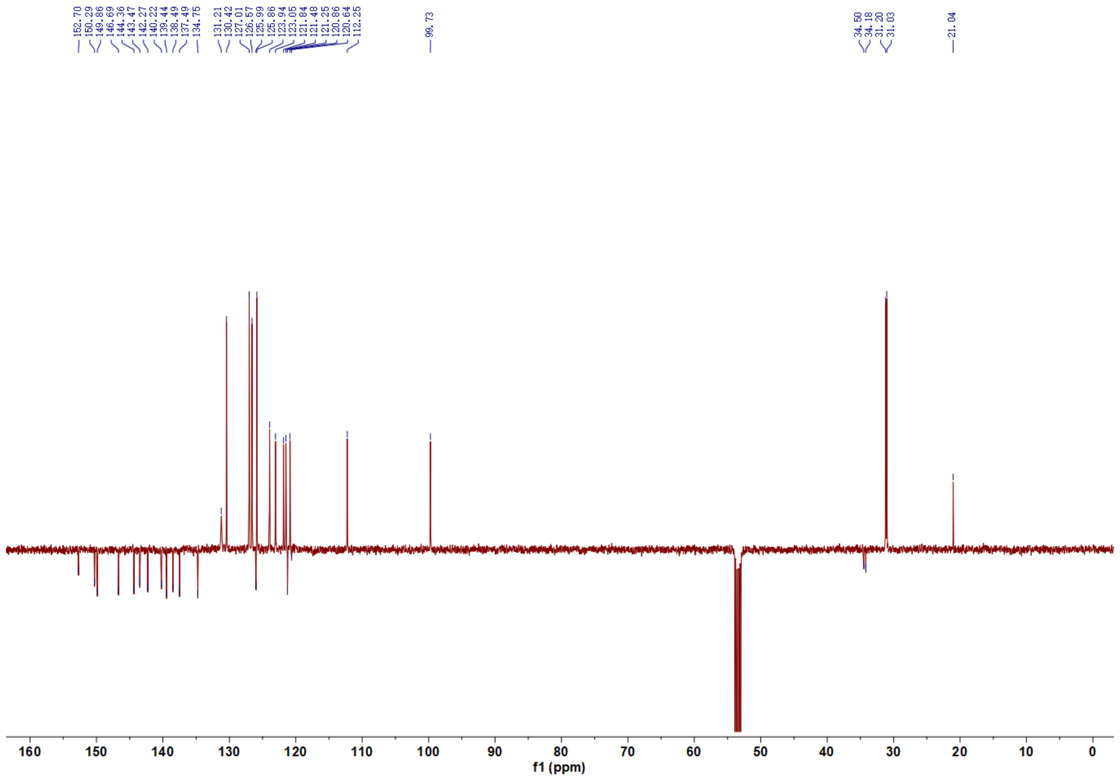

Figure **S20**. DEPTQ ^{13}C NMR spectrum of **DPA-tBuPh-BN** in CD_2Cl_2.

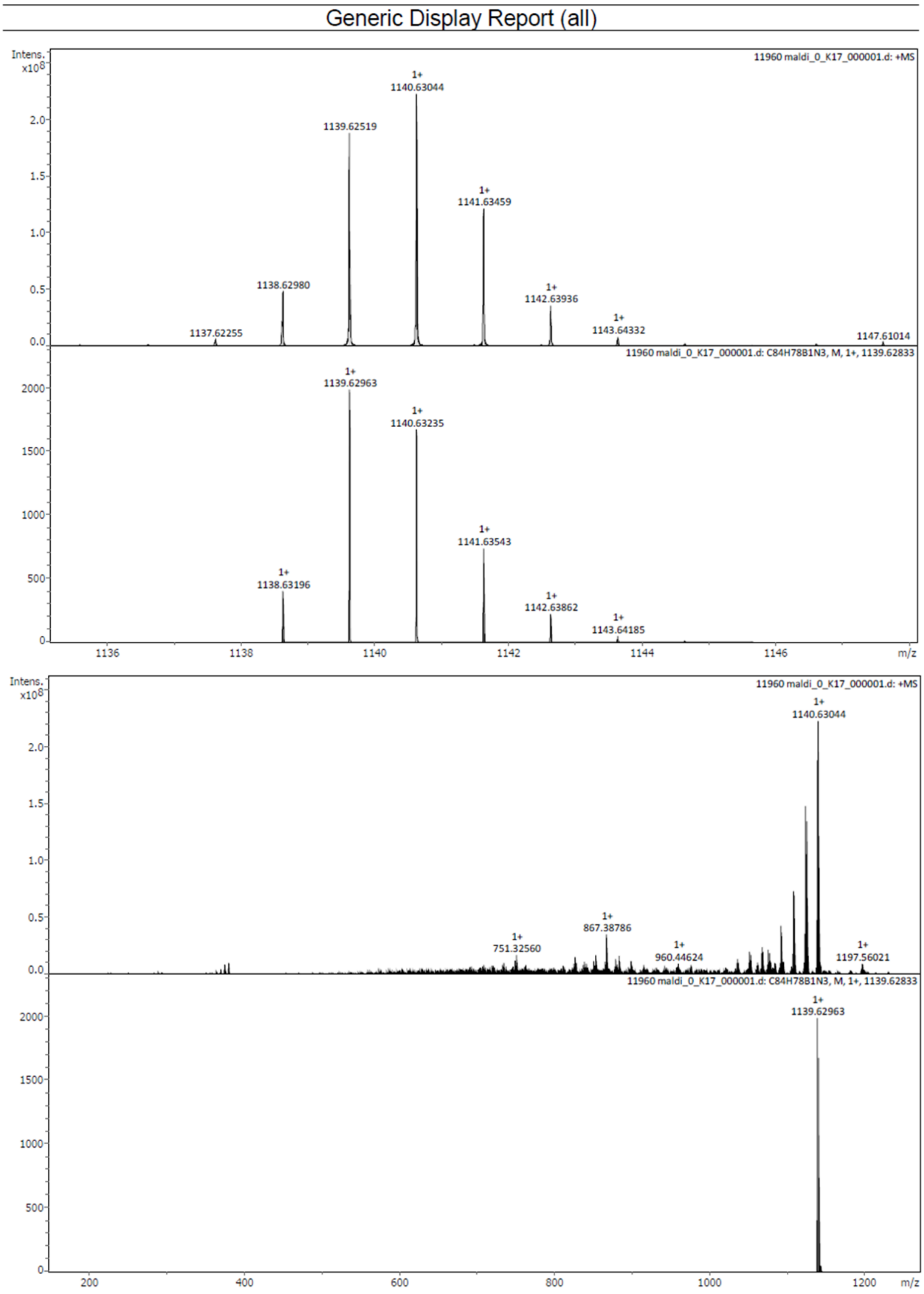

Figure **S21**. HRMS spectrum of **DPA-tBuPh-BN**.

LONDON METROPOLITAN UNIVERSITY
HUMAN SCIENCES

Elemental Analysis Sample Results

**Name**	Jingxiang Wang
**Organisation Name**	University of St Andrews
**Purchase order number**	

**Standard – Acetanilide**		
**Element**	**Expected %**	**Found**
**Carbon**	71.10 (+/- 0.23)	71.02
**Hydrogen**	6.71 (+/- 0.07)	6.64
**Nitrogen**	10.34 (+/- 0.09)	10.29

**Analysis – DPA-tBuPh-BN**			
**Element**	**Expected %**	**Found (1)**	**Found (2)**
**Carbon**	88.47	88.47	88.25
**Hydrogen**	6.89	6.78	6.96
**Nitrogen**	3.68	3.47	3.39

**Date completed**	16.04.2024
**Signature**	O. McCullough
**Comments**	

Figure **S22**. Elemental analysis data of **DPA-tBuPh-BN**.

HPLC Trace Report02Aug2023

<Sample Information>

Sample Name	: dpatbuphbn		
Sample ID	:		
Method Filename	: 80% THF 20% water 0.6 mlmin 20 mins.lcm		
Batch Filename	: 01082023.lcb		
Vial #	: 2-9	Sample Type	: Unknown
Injection Volume	: 5 uL		
Date Acquired	: 01/08/2023 18:33:52	Acquired by	: System Administrator
Date Processed	: 01/08/2023 18:53:55	Processed by	: System Administrator

<Chromatogram>

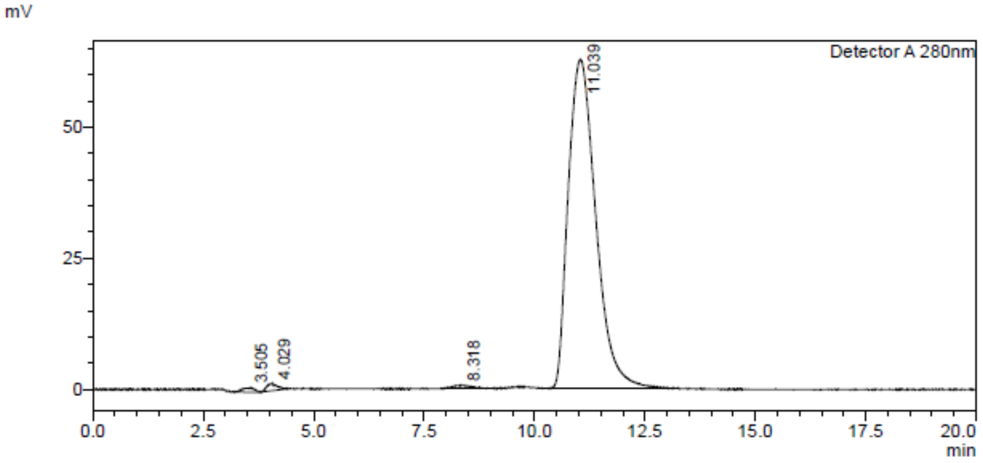

<Peak Table>

Detector A 280nm

Peak#	Ret. Time	Area	Height	Area%	Area/Height	Width at 5% Height
1	3.505	15630	773	0.564	20.224	0.532
2	4.029	23445	1310	0.847	17.895	0.567
3	8.318	16841	546	0.608	30.819	0.915
4	11.039	2712929	62662	97.981	43.295	1.426
Total		2768845	65291	100.000		

Figure **S23**. HPLC spectrum of **DPA-tBuPh-BN**.

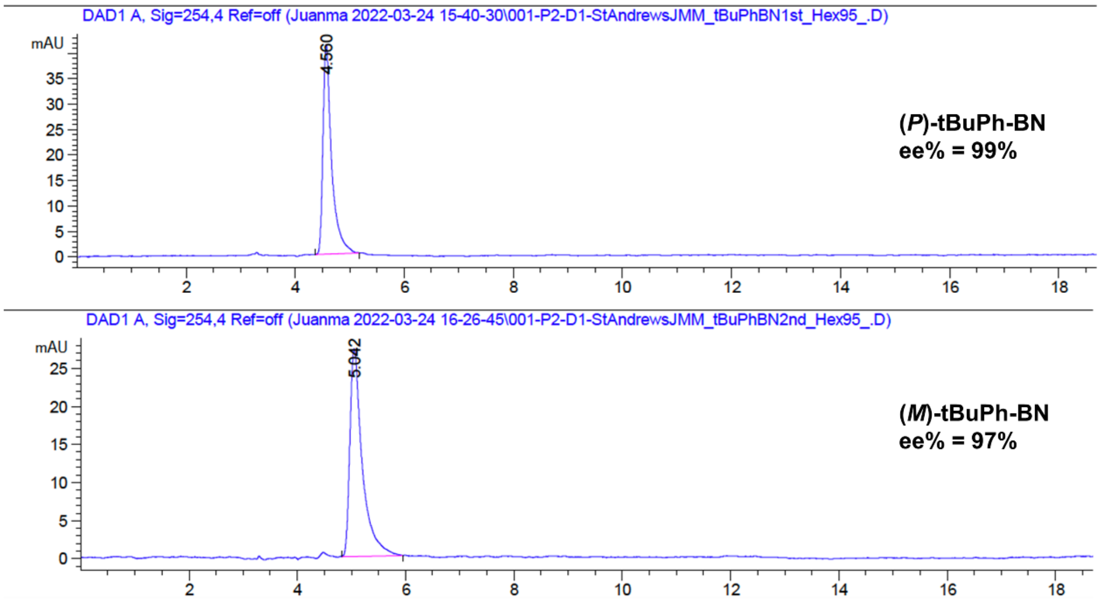

Figure **S24**. Chiral HPLC spectra of (*P*)-**tBuPh-BN** and (*M*)-**tBuPh-BN** (Column: CHIRALPAK IE; Solvent system: Hexane:THF 95:5; Detector: 254 nm).

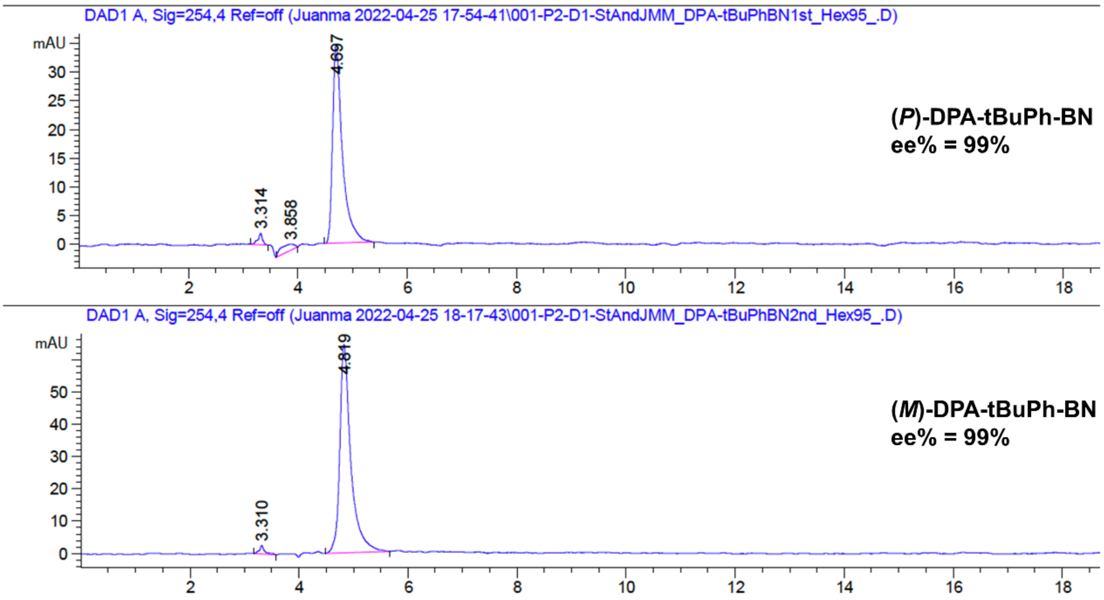

Figure **S25**. Chiral HPLC spectra of (*P*)-**DPA-tBuPh-BN** and (*M*)-**DPA-tBuPh-BN** (Column: CHIRALPAK IE; Solvent system: Hexane:THF 95:5; Detector: 254 nm).

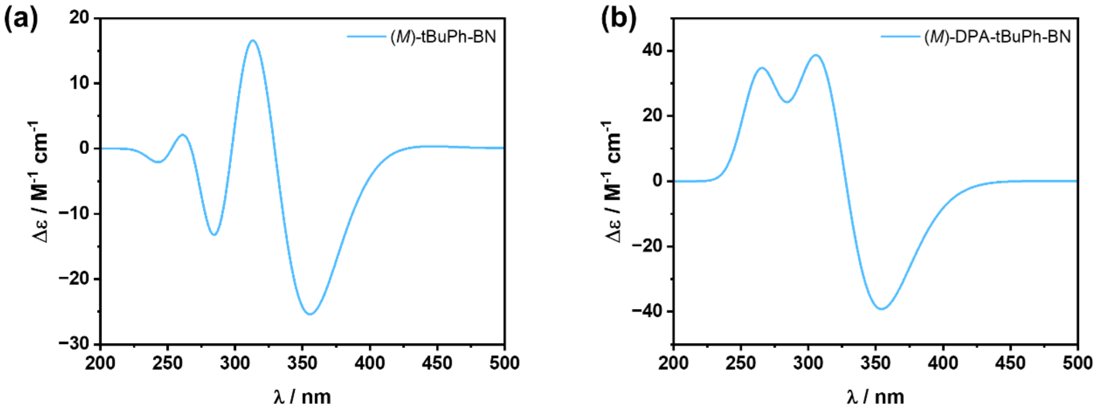

Figure **S26**. Theoretically simulated CD spectra of (*M*)-**tBuPh-BN** and (*M*)-**DPA-tBuPh-BN** calculated at the TDA-DFT/M062X/6-31G(d,p) in toluene.

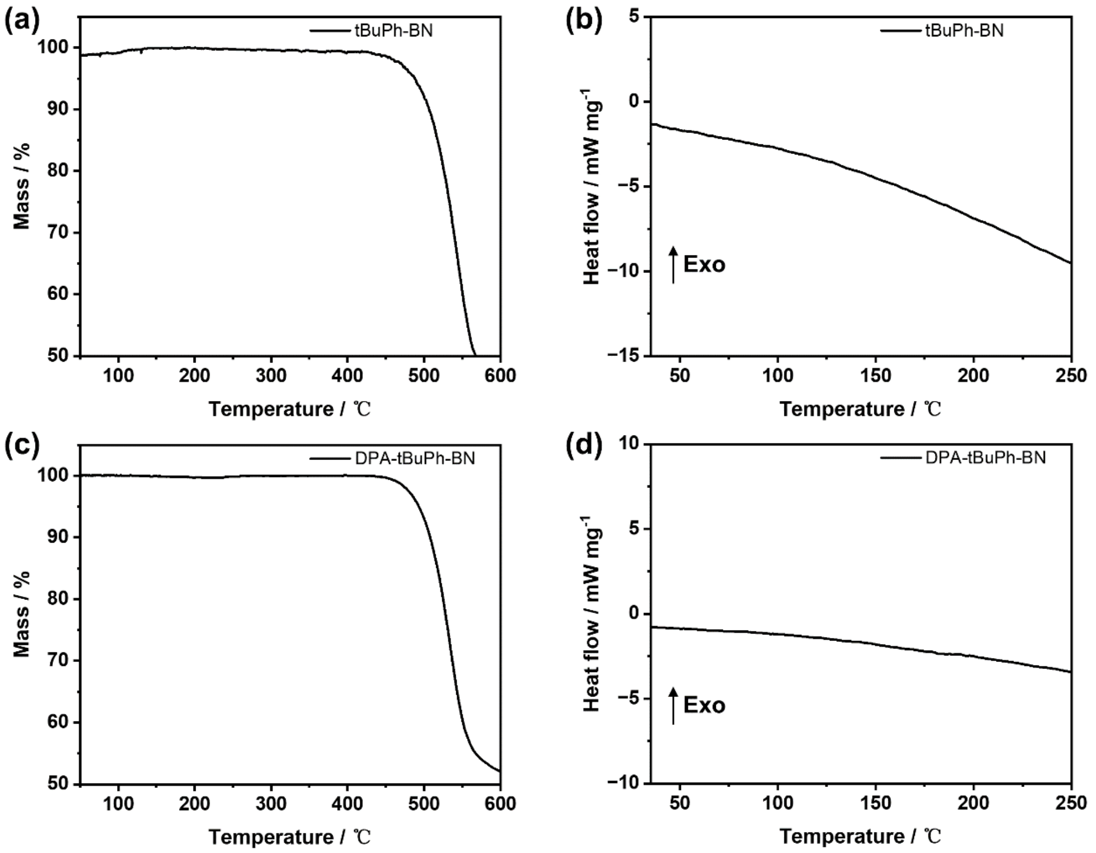

Figure **S27**. (a) TGA and (b) DSC analyses of **tBuPh-BN** and (c) TGA and (d) DSC analyses of **DPA-tBuPh-BN**.

X-ray Crystallography

X-ray diffraction data for compound **tBuPh-BN** were collected using a Rigaku FR-X Ultrahigh Brilliance Microfocus RA generator/confocal optics with XtaLAB P200 diffractometer [Mo Kα radiation (λ = 0.71073 Å)]. Diffraction data for compound **DPA-tBuPh-BN** were collected using a Rigaku MM-007HF High Brilliance RA generator/confocal optics [Cu Kα radiation (λ = 1.54187 Å)] with XtaLAB P200 diffractometer. Data for both compounds were collected at 173 K using either CrystalClear[26] (using ω steps and accumulating area detector images spanning at least a hemisphere of reciprocal space) or CrysAlisPro[27] (using a calculated strategy), and processed (including correction for Lorentz, polarization and absorption) using CrysAlisPro. Structures were solved by dual-space methods (SHELXT[28]) and refined by full-matrix least-squares against F^2 (SHELXL-2019/3[29]). Non-hydrogen atoms were refined anisotropically (except as specified below), and hydrogen atoms were refined using a riding model except for the solvent OH hydrogens in **DPA-tBuPh-BN** which were located from the difference Fourier map and refined isotropically with a distance restraint, and with thermal motion riding on that of the parent atom. The structure of **tBuPh-BN** showed high proportions of void space (771 $Å^3$) and the SQUEEZE[30] routine implemented in PLATON[31] was used to remove the contribution to the diffraction pattern of the unordered electron density in the void spaces. The structure of **DPA-tBuPh-BN** showed rotational disorder in the orientation of one *t*Bu group which was modelled over two sites with restraints to bond distances and thermal motion. Atoms in the minor component of the disorder were refined isotropically. The structure also showed disorder in the orientation of the MeOH solvent molecule, which was also modelled over two sites with restraints to bond distances. The structure model of this compound showed it in the non-centrosymmetric space group $P2_12_12_1$, with a refined Flack parameter of 0.04(5), indicative of a majority of a single enantiomer, in this case the *M*-enantiomer. All calculations except SQUEEZE were performed using the Olex2[32] interface.

Selected crystallographic data are presented in Table **S1**. CCDC 2356677 and 2356678 contains the supplementary crystallographic data for this paper. These data can be obtained free of charge from The Cambridge Crystallographic Data Centre via www.ccdc.cam.ac.uk/structures.

Table **S1**. Selected crystallographic data.

	**tBuPh-BN**	**DPA-tBuPh-BN**
formula	$C_{70}H_{65}BN_2$	$C_{89}H_{90}BN_3O_2$
fw	945.05	1244.44
crystal description	yellow prism	yellow prism
crystal size [mm^3]	0.20×0.10×0.07	0.25×0.12×0.03
space group	$P\bar{1}$	$P2_12_12_1$
Flack parameter	-	0.04(5)
a [Å]	13.4271(5)	10.53694(4)
b [Å]	13.9487(4)	20.05694(9)
c [Å]	19.3551(8)	33.47918(14)
α [°]	102.551(3)	
β [°]	105.385(4)	
γ [°]	106.129(3)	
vol [Å]^3	3187.5(2)	7075.45(5)
Z	2	4
ρ (calc) [g/cm^3]	0.985	1.168
μ [mm^{-1}]	0.056	0.522
F(000)	1008.0	2664
reflections collected	61542	85843
independent reflections (R_{int})	15033 (0.0498)	14502 (0.0206)
parameters, restraints	670, 0	913, 87
GooF on F^2	1.027	1.043
R_1 [$I > 2\sigma(I)$]s	0.0983	0.0371
wR_2 (all data)	0.3208	0.1038
largest diff. peak/hole [e/Å^3]	0.49/-0.24	0.284, -0.233

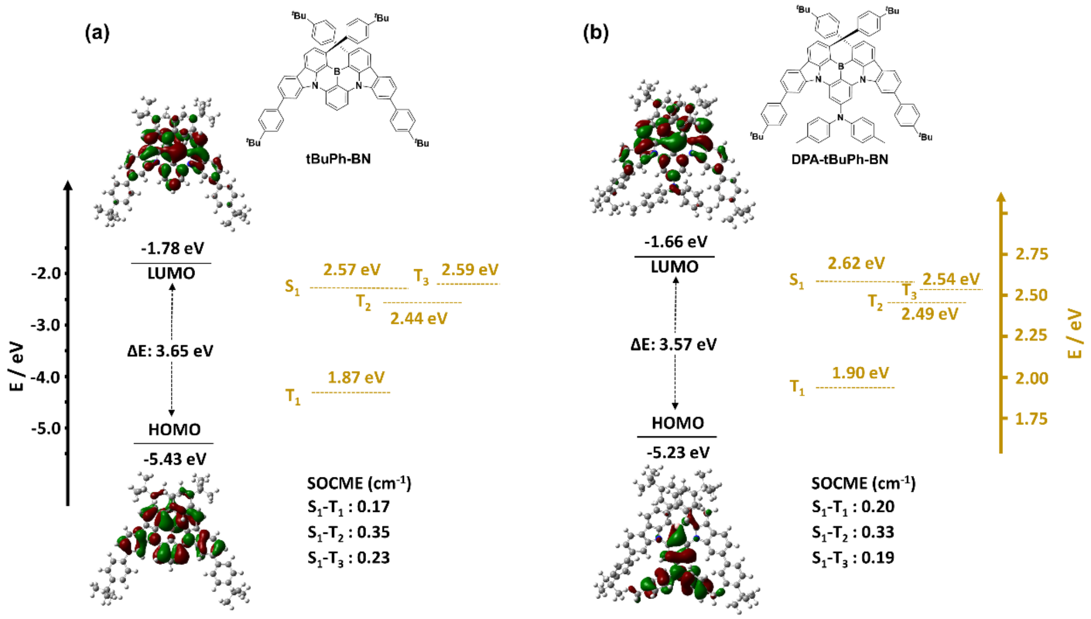

Figure **S28**. Calculated electron density distribution (isovalue: 0.02) of the HOMO, LUMO and energy levels in the gas phase based on optimized ground-state geometries and SOCME based on optimized T_1 geometries at the PBE0/6-31G(d,p) level.

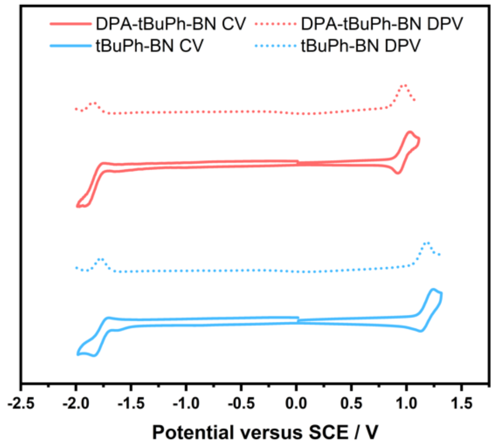

Figure **S29**. CV and DPV of **tBuPh-BN** and **DPA-tBuPh-BN** in degassed DCM with 0.1 M $[^{n}Bu_4N]PF_6$ as the supporting electrolyte and Fc/Fc^+ as the internal reference versus SCE (0.46 V vs. SCE)[19]. CV was performed at a sweep rate of 100 mV/s. DPV was conducted with an increment potential of 0.01 V and a pulse amplitude, width, and period of 50 mV, 0.06, and 0.5 s, respectively.

Table **S2**. Electrochemical data of **tBuPh-BN** and **DPA-tBuPh-BN**.[a]

	E_{ox} / V	E_{red} / V	HOMO/ eV	LUMO/ eV	ΔE / eV
tBuPh-BN	0.73	-2.23	-5.53	-2.57	2.96
DPA-tBuPh-BN	0.51	-2.31	-5.31	-2.49	2.82

[a] In degassed DCM with 0.1 M [^{n}Bu$_4$N]PF$_6$ as the supporting electrolyte and Fc/Fc$^+$ as the internal reference (0.46 V vs. SCE)[19]. The HOMO and LUMO energies were determined using the relation HOMO/LUMO = -(E_{ox} / E_{red} + 4.8) eV,[21] where E_{ox} and E_{red} are the peak of anodic and cathodic potentials from DPV relative to Fc/Fc$^+$. ΔE is the energy gap between HOMO and LUMO.

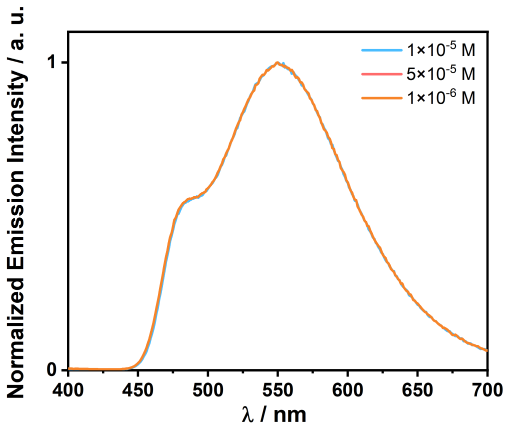

Figure **S30**. PL spectra of **DPA-tBuPh-BN** in acetone at different concentrations at 300 K. (λ_{exc} = 340 nm).

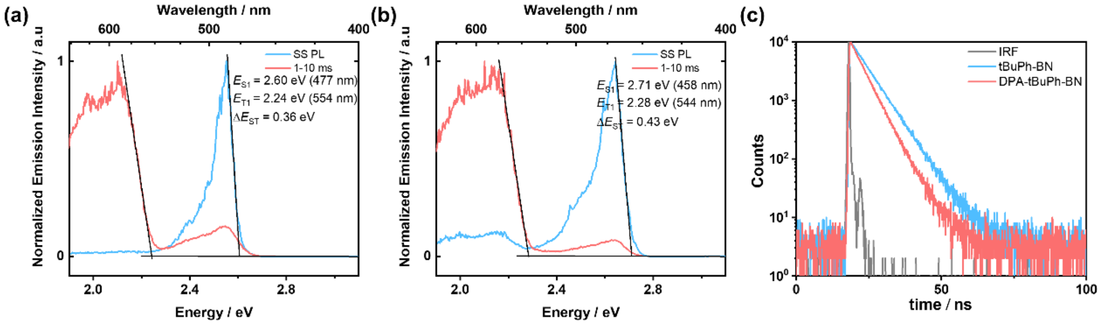

Figure **S31**. Steady-state PL and phosphorescence spectra of (a) **tBuPh-BN** and (b) **DPA-tBuPh-BN** in 2-MeTHF at 77 K (λ_{exc} = 340 nm). (c) Time-resolved PL decay of the prompt emission in degassed toluene (λ_{exc} = 375 nm).

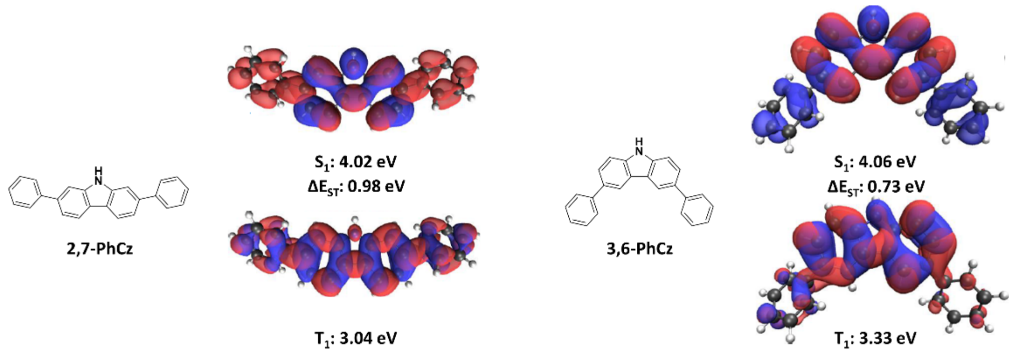

Figure **S32**. Density plot of the natural transition orbitals (NTOs) (hole (blue) & electron (red)) of the S_1 and T_1 states for **2,7-PhCz** and **3,6-PhCz**, plotted with isovalue: 0.02.

Table **S3**. Φ_{PL} values of **tBuPh-BN** and **DPA-tBuPh-BN** in SF3-TRZ films (λ_{exc} = 420 nm).

Φ_{PL} (λ_{PL}) / % (nm)	1 wt% in SF3-TRZ	2 wt% in SF3-TRZ	4 wt% in SF3-TRZ	10 wt% in SF3-TRZ
tBuPh-BN	67 (490)	63 (496)	55 (495)	42 (496)
DPA-tBuPh-BN	47 (484)	46 (487)	36 (487)	30 (491)

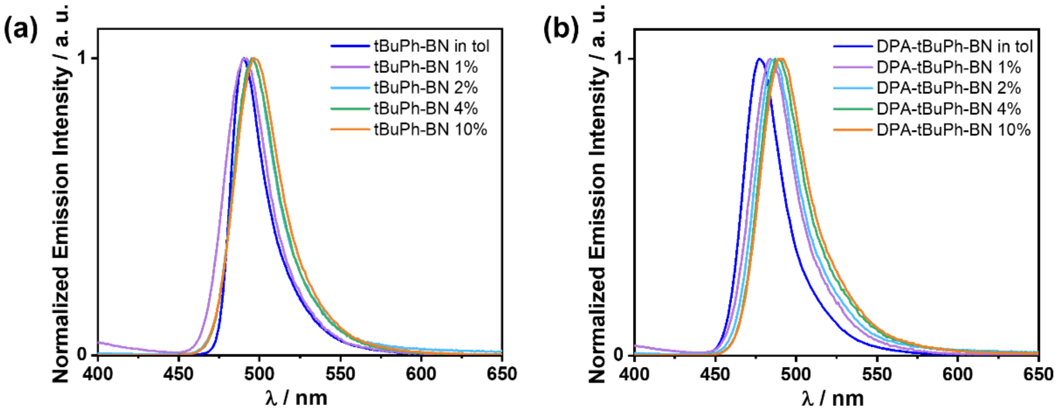

Figure **S33**. Steady-state PL spectra of (a) **tBuPh-BN** and (b) **DPA-tBuPh-BN** at 300 K in toluene and different doping concentration films in SF3-TRZ (λ_{exc} = 340 nm).

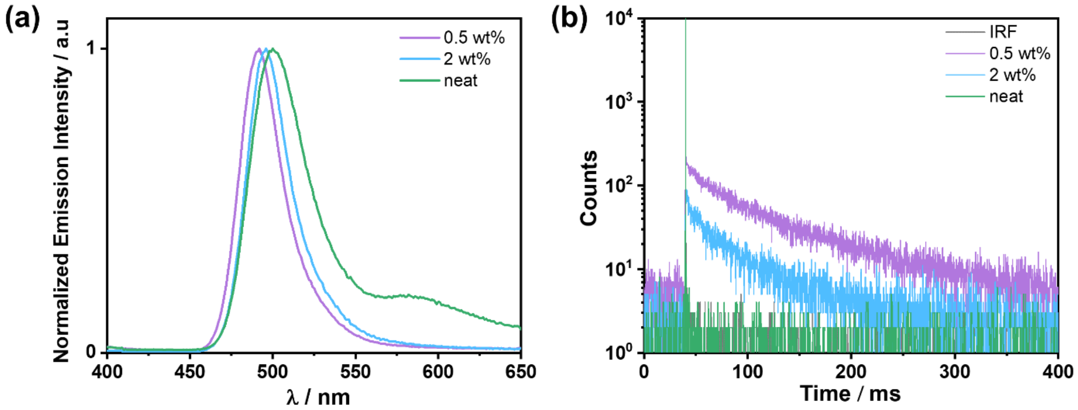

Figure **S34**. (a) Steady-state PL spectra and (b) time-resolved PL decay of the 0.5, 2 wt% doped films in SF3-TRZ and neat film of **tBuPh-BN** (λ_{exc} = 340 nm).

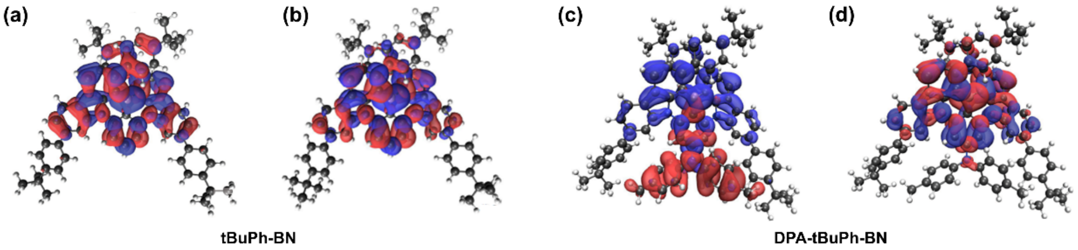

Figure **S35**. Density plot of the NTO hole (blue) & electron (red) of the S_1 states for **tBuPh-BN** calculated at (a) PBE0, (b) M062X and **DPA-tBuPh-BN** calculated at (c) PBE0, (d) M062X, plotted with isovalue: 0.02.

Table **S4**. Summary of the calculated chiroptical properties of **tBuPh-BN** and **DPA-tBuPh-BN** at the optimized S_1 geometry.

$S_1 - S_0$	$\lvert\boldsymbol{\mu}\rvert$ / 10^{-20} esu·cm	$\lvert\boldsymbol{m}\rvert$ / 10^{-20} erg/Gauss	θ / °	$\cos\theta$	g_{cal}
(*M*)-**tBuPh-BN**	539	1.22	100	-0.18	-1.6×10^{-3}
(*M*)-**DPA-tBuPh-BN**	551	0.726	97	-0.13	-0.67×10^{-3}

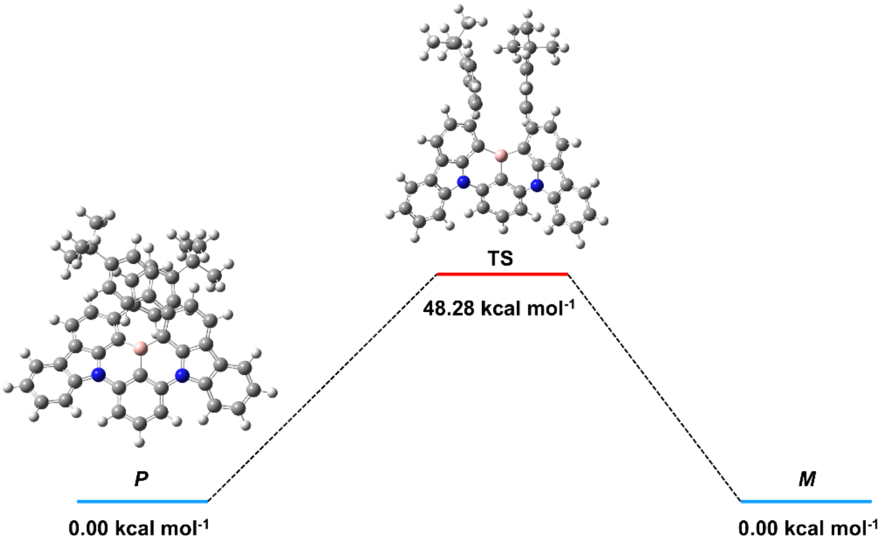

Figure **S36**. Calculated racemization energy barrier of the helicene moiety at the M062X/6-31G(d,p) level.

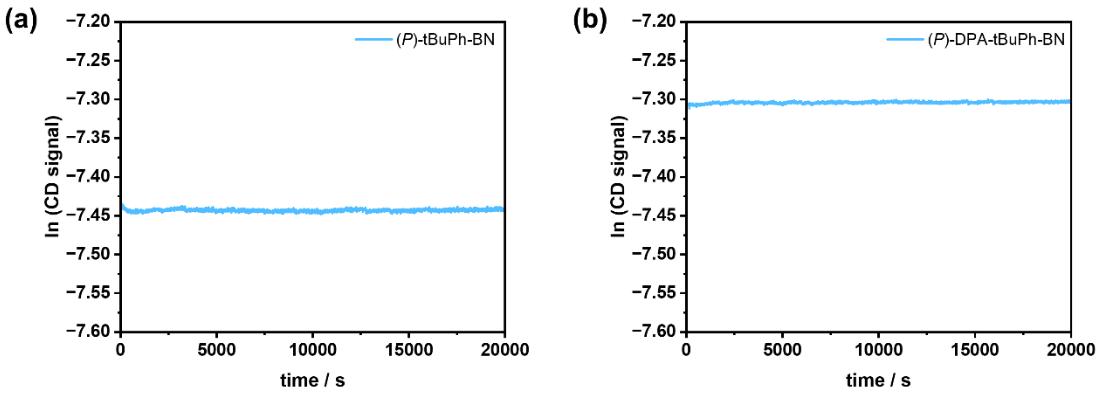

Figure **S37**. CD signal time evolution of (*P*)-**tBuPh-BN** and (*P*)-**DPA-tBuPh-BN** in toluene solutions measured at 330 nm and 90 °C for 20000 s.

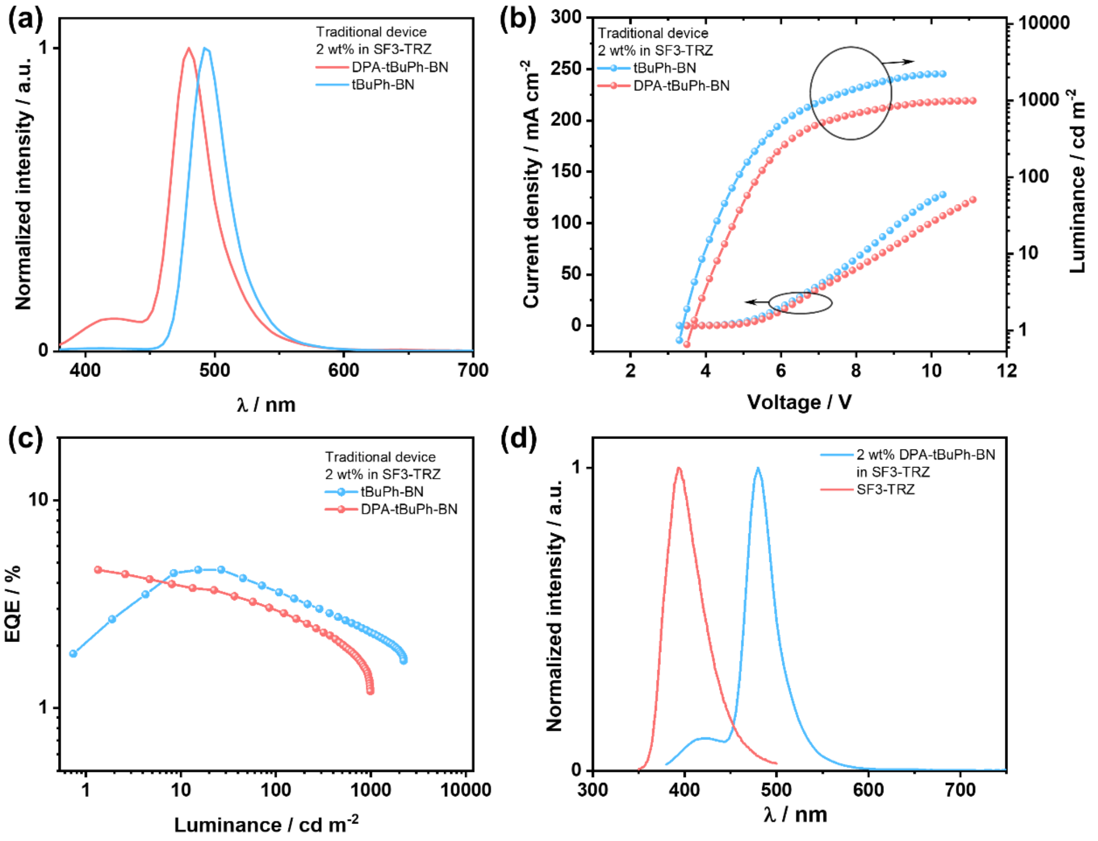

Figure **S38**. (a) EL spectra; (b) *JVL* characteristics; (c) EQE vs luminescence for the OLED with 2 wt% emitter doped in SF3-TRZ host in the EML; (d) A comparison of the PL spectra of SF3-TRZ in neat film and the 2 wt% emitter doped film in SF3-TRZ.

Table **S5**. Device data with an EML consisting of 2 wt% **tBuPh-BN** and **DPA-tBuPh-BN** in SF3-TRZ.

	V_{on} / V	λ_{EL} (FWHM) / nm	L_{max} / cd m^{-2}	$EQE_{max/100/1000}$ / %	CIE (x, y)
tBuPh-BN	3.4	492 (33)	2217	4.6/3.6/2.3	0.10, 0.45
DPA-tBuPh-BN	3.6	480 (35)	990	4.6/2.9/-	0.12, 0.22

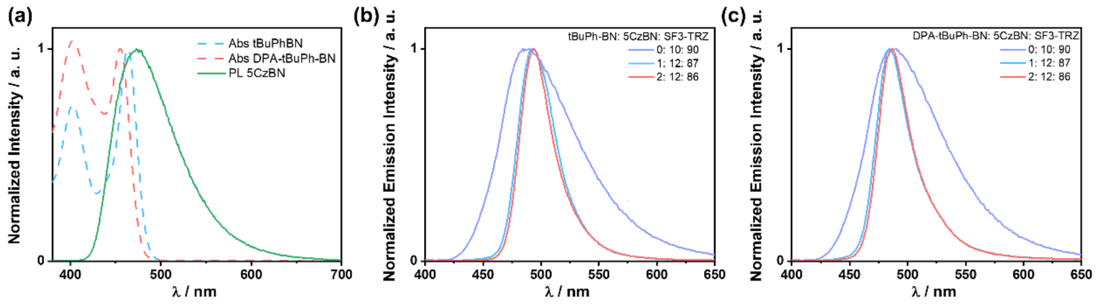

Figure **S39**. (a) UV/vis absorption spectra of **tBuPh-BN** and **DPA-tBuPh-BN** and PL spectrum of **5CzBN** in toluene (λ_{exc} = 340 nm). PL spectra of different doping concentration films of (b) **tBuPh-BN** and (c) **DPA-tBuPh-BN** with **5CzBN** in SF3-TRZ host.

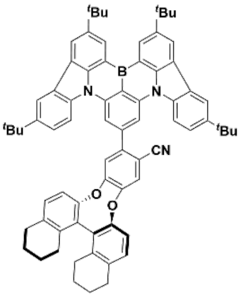

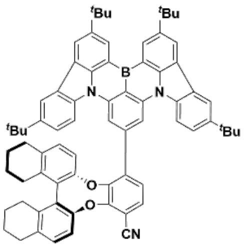

OBN-2CN-BN **OBN-4CN-BN**

Adv. Mater. **2021**, *33*, 2100652

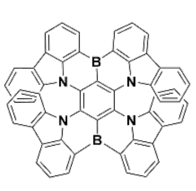

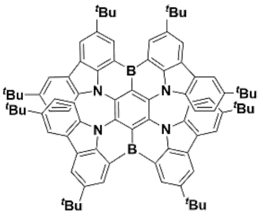

R-BN **R-TBN**

Angew. Chem. Int. Ed. **2021**, *60*, 20498
J. Am. Chem. Soc. **2021**, *143*, 17958

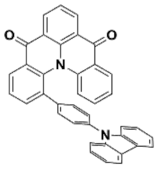

QAO-PhCz

Chem. Commun. **2021**, *57*, 11041

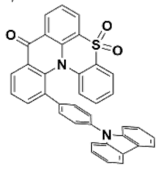

QPO-PhCz

J. Mater. Chem. C **2022**, *10*, 4393

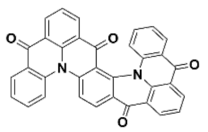

Hel-DiDiKTa

J. Mater. Chem. C **2022**, *10*, 4861

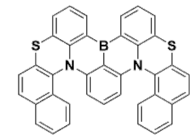

Helicene-BN

CCS Chem. **2022**, *4*, 3463

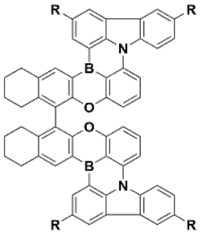

R = H R-DOBN
R = ^{t}Bu R-DOBNT

Adv. Mater. **2022**, *34*, 2204253

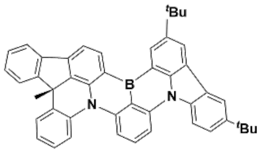

BN-MeIAc

Angew. Chem. Int. Ed. **2022**, *61*, e202202227

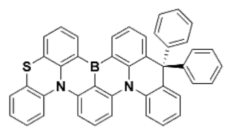

BN4

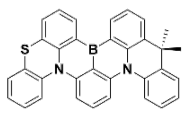

BN5

Adv. Mater. **2022**, *34*, 2105080

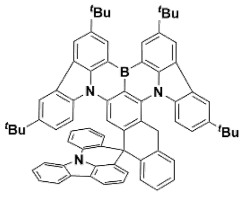

Spiro-BNCz

Aggregate **2023**, *5*, e445

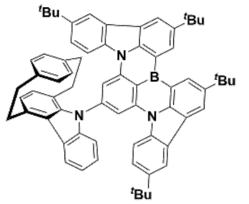

Czp-tBuCzB

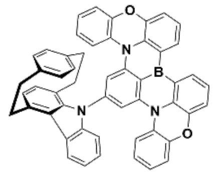

Czp-POAB

Angew. Chem. Int. Ed. **2023**, *62*, e202217045

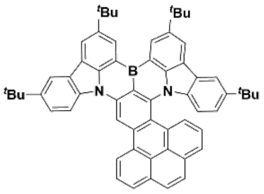

BN-Py

Adv. Mater. **2023**, *35*, 2305125

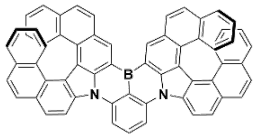

3-C$_2$

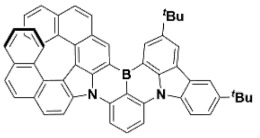

4-C$_1$

Angew. Chem.Int. Ed. **2023**, *62*, e202218965

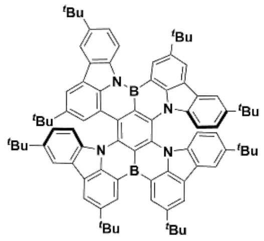

RBNN

Adv. Mater. **2024**, *36*, 2307420

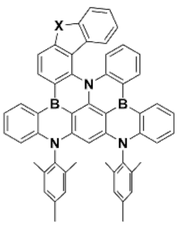

X = O DB-O
X = S DB-S

Adv. Mater. **2024**, *36*, 2308314

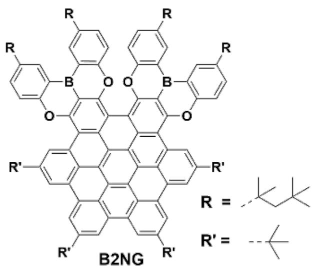

B2NG

Chem. Eur. J. **2024**, *30*, e202304169

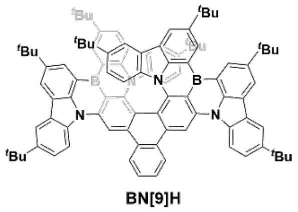

BN[9]H

Angew. Chem. Int. Ed. **2024**, e202401835

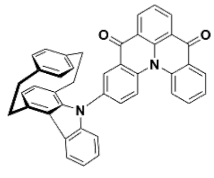

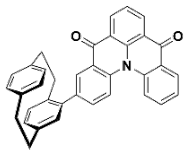

PCP-DiKTa **Czp-DiKTa**

Adv. Funct. Mater. **2024**, 2402036

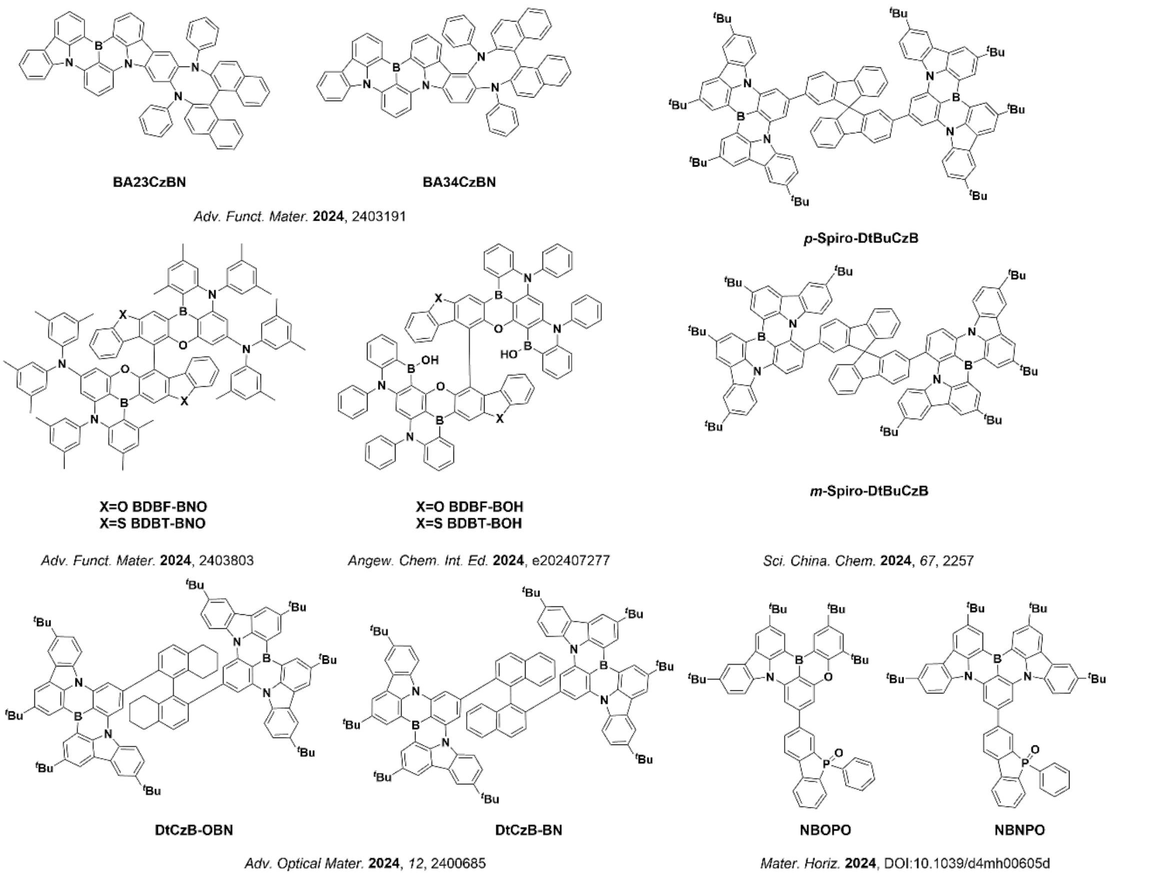

Figure **S40**. The structures of chiral MR-TADF emitters.

Table **S6**. Properties of chiral MR-TADF emitters and OLEDs.

Emitters	λ_{PL} (FWHM) / nm	Φ_{PL} / %	$\lvert g_{PL} \rvert$	λ_{EL} (FWHM) / nm	EQE_{max} / %	EQE_{100} / %	Ref.
OBN-2CN-BN*	498 (32) [a]	95 [a]	9.1×10^{-4} [a]	496 (33)	29.8	27.2	33
OBN-4CN-BN*	510 (35) [a]	90 [a]	1.04×10^{-3} [a]	508 (34)	24.7	23.5	
R-BN	660 (38) [b]	100 [b]	2×10^{-3} [c]	664 (48)	28.1	-	34, 35
R-TBN	692 (38) [b]	100 [b]	2×10^{-3} [c]	686 (49)	27.6	-	
QAO-PhCz	461 (29) [b]	47 [g]	1.1×10^{-3} [b]	467 (36)	14	-	36
QPO-PhCz	446 (58) [b]	51 [h]	1.6×10^{-3} [c]	488 (-)	10.6	-	37
Hel-DiDiKTa	478 (52) [i]	4.1 [i]	4×10^{-4} [b]	-	-	-	38
Helicene-BN	525 (48) [d]	98 [d]	2.1×10^{-3} [b]	523 (49)	31.5	29.6	39
R-DOBN	453 (21) [b]	91 [e]	1×10^{-3} [e]	459 (38)	23.9	12.5	40

R-DOBNT	459 (21) [b]	96 [e]	0.9×10^{-3} [e]	464 (35)	25.6	13.9	
BN-MeIAc	497 (30) [b]	96 [d]	3.2×10^{-4} [d]	504 (33)	37.2	36.1	41
BN4	500 (43) [b]	88 [b]	(1-2) $\times 10^{-3}$ [b]	510 (49)	20.6	20.5	42
BN5	497 (43) [b]	87 [b]	(1-2) $\times 10^{-3}$ [b]	506 (48)	26.5	17.6	
Spiro-BNCz	528 (42) [b]	92 [e]	1.3×10^{-3} [e]	533 (48)	34.2	22.5	43
Czp-tBuCzB	478 (23) [b]	98 [e]	1.6×10^{-3} [e]	479 (24)	32.1	29.2	44
Czp-POAB	497 (36) [b]	96 [e]	1.4×10^{-3} [e]	513 (48)	28.7	28.1	
BN-Py*	527 (35) [b]	90 [b]	3.67×10^{-4} [a.]	532 (38)	31.0	30.8	45
3-C_2	543 (23) [b]	72 [b]	1.5×10^{-3} [b]	-	-	-	46
4-C_1	520 (28) [b]	85 [b]	1.1×10^{-3} [b]	-	-	-	
RBNN	617 (38) [b]	96 [b]	1.41×10^{-3} [b]	617 (48)	36.6	26.7	47
DB-O	443 (24) [b]	91 [f]	1.4×10^{-3} [b]	445 (24)	27.5	21.3	48
DB-S	444 (23) [b]	95 [f]	1.5×10^{-3} [b]	447 (24)	29.3	23.6	
B2NG	588 [b]	50 [b]	2.5×10^{-3} [b]	-	-	-	49
BN[9]H	578 (47) [b]	98 [b]	5.8×10^{-3} [b]	580 (48)	36.7	33.4	50
PCP-DiKTa	469 (37) [b]	93 [j]	-	489 (53)	25.7	19.1	51
Czp-DiKTa	505 (66) [b]	99 [j]	4×10^{-4} [b]	518 (69)	29.2	25.9	
BDBF-BNO	460 (27) [b]	93 [k]	1.7×10^{-3} [b]	473 (38)	32.1	20.8	52
BDBT-BNO	460 (27) [b]	96 [k]	1.8×10^{-3} [b]	470 (34)	35.7	26.8	
BDBF-BOH	458 (27) [b]	90 [k]	6.8×10^{-4} [b]	467 (30)	29.5	15.8	53
BDBT-BOH	459 (27) [b]	91 [k]	8.5×10^{-4} [b]	467 (30)	30.1	19.7	
DtCzB-OBN	491 (22) [b]	97 [e]	6.5×10^{-4} [e]	493 (22)	30.0	-	54
DtCzB-BN	494 (22) [b]	98 [e]	1.8×10^{-3} [e]	494 (22)	33.9	-	
p-Spiro-DtBuCzB	491 (25) [b]	95 [e]	3.3×10^{-4} [b]	498	29.6	-	55
m-Spiro-DtBuCzB	502 (33) [b]	96.7 [e]	6.6×10^{-4} [b]	508	33.8	-	
NBOPO	462 (25) [b]	87 [e]	1.2×10^{-3} [e]	468 (31)	16.4	2.51	56
NBNPO	498 (24) [b]	93 [e]	4.3×10^{-3} [e]	502 (29)	28.3	14.1	
**tBuPh-BN***	490 (25) [b]	85 [b]	1.5×10^{-3} [b]	492 (34)	20.9	9.8	

**DPA-tBuPh-BN***	477 (28) [b]	54 [b]	0.9×10^{-3} [b]	480 (38)	15.9	10.1	**This work**

[a.] 3 wt% doped films in PhCzBCz; [b] In toluene; [c] In DCM; [d.] 1 wt% doped films in DMIC-TRZ; [e] 5 wt% doped films in 2,6-DCzPPy; [f] 2 wt% doped films in DOBNA-OAr; [g] 5 wt% doped films in mCBP; [h] 18 wt% doped films in DPEPO; [i] 1 wt% doped films in mCP; [j] 2 wt% doped films in 2,6-DCzPPy; [k] 10 wt% doped films in 2,6-DCzPPy;* HF devices.

References:

1. M. Frisch, G. Trucks, H. Schlegel, G. Scuseria, M. Robb, J. Cheeseman, G. Scalmani, V. Barone, B. Mennucci and G. Petersson, *Gaussian 09 Revision D. 01*, 2009.
2. C. Adamo and V. Barone, Toward reliable density functional methods without adjustable parameters: The PBE0 model, *J. Chem. Phys.*, 1999, **110**, 6158-6170.
3. T. H. Dunning, Jr., Gaussian basis sets for use in correlated molecular calculations. I. The atoms boron through neon and hydrogen, *J. Chem. Phys.*, 1989, **90**, 1007-1023.
4. S. Grimme, Density functional calculations with configuration interaction for the excited states of molecules, *Chem. Phys. Lett.*, 1996, **259**, 128-137.
5. S. Hirata and M. Head-Gordon, Time-dependent density functional theory within the Tamm–Dancoff approximation, *Chem. Phys. Lett.*, 1999, **314**, 291-299.
6. H. Kubo, T. Hirose, T. Nakashima, T. Kawai, J.-y. Hasegawa and K. Matsuda, Tuning transition electric and magnetic dipole moments:[7] helicenes showing intense circularly polarized luminescence, *J. Phys. Chem. Lett.*, 2021, **12**, 686-695.
7. R. Dennington, T. Keith and J. Millam, *GaussView, Version 6*, Semichem Inc., Shawnee Mission KS, 2019.
8. C. Hättig, Geometry optimizations with the coupled-cluster model CC2 using the resolution-of-the-identity approximation, *J. Chem. Phys.*, 2003, **118**, 7751-7761.
9. A. Hellweg, S. A. Grün and C. Hättig, Benchmarking the performance of spin-component scaled CC2 in ground and electronically excited states, *Phys. Chem. Chem. Phys.*, 2008, **10**, 4119-4127.
10. K. Momma and F. Izumi, VESTA 3 for three-dimensional visualization of crystal, volumetric and morphology data, *J. Appl. Crystallogr.*, 2011, **44**, 1272-1276.

11. O. S. Lee and E. Zysman-Colman, *Silico (version 3.1), In-Silico-Computing*, St Andrews, Scotland, 2023.

12. N. M. O'Boyle, A. L. Tenderholt and K. M. Langner, Cclib: a library for package ‐ independent computational chemistry algorithms, *J. Comput. Chem.*, 2008, **29**, 839-845.

13. W. Humphrey, A. Dalke and K. Schulten, VMD: visual molecular dynamics, *J. Mol. Graph. Model.*, 1996, **14**, 33-38.

14. J. E. Stone, *An efficient library for parallel ray tracing and animation*, 1998.

15. J. D. Hunter, Matplotlib: A 2D graphics environment, *Comput. Sci. Eng.*, 2007, **9**, 90-95.

16. N. M. O'Boyle, M. Banck, C. A. James, C. Morley, T. Vandermeersch and G. R. Hutchison, Open Babel: An open chemical toolbox, *J. Cheminformatics*, 2011, **3**, 1-14.

17. N. M. O'Boyle, C. Morley and G. R. Hutchison, Pybel: a Python wrapper for the OpenBabel cheminformatics toolkit, *Chem. Cent. J.*, 2008, **2**, 1-7.

18. X. Gao, S. Bai, D. Fazzi, T. Niehaus, M. Barbatti and W. Thiel, Evaluation of spin-orbit couplings with linear-response time-dependent density functional methods, *J. Chem. Theory Comput.*, 2017, **13**, 515-524.

19. N. G. Connelly and W. E. Geiger, Chemical redox agents for organometallic chemistry, *Chem. Rev.*, 1996, **96**, 877-910.

20. J. Pommerehne, H. Vestweber, W. Guss, R. F. Mahrt, H. Bässler, M. Porsch and J. Daub, Efficient two layer leds on a polymer blend basis, *Adv. Mater.*, 1995, **7**, 551-554.

21. C. M. Cardona, W. Li, A. E. Kaifer, D. Stockdale and G. C. Bazan, Electrochemical Considerations for Determining Absolute Frontier Orbital Energy Levels of Conjugated Polymers for Solar Cell Applications, *Adv. Mater.*, 2011, **23**, 2367-2371.

22. G. A. Crosby and J. N. Demas, Measurement of photoluminescence quantum yields. Review, *J. Phys. Chem.*, 1971, **75**, 991-1024.

23. W. H. Melhuish, Quantum efficiencies of fluorescence of organic substances: effect of solvent and concentration of the fluorescent solute1, *J. Phys. Chem.*, 1961, **65**, 229-235.

24. N. Greenham, I. Samuel, G. Hayes, R. Phillips, Y. Kessener, S. Moratti, A. Holmes and R. Friend, Measurement of absolute photoluminescence quantum efficiencies in conjugated polymers, *Chem. Phys. Lett.*, 1995, **241**, 89-96.
25. F. Zinna, T. Bruhn, C. A. Guido, J. Ahrens, M. Bröring, L. Di Bari and G. Pescitelli, Circularly polarized luminescence from axially chiral BODIPY DYEmers: an experimental and computational study, *Chem. Eur. J.*, 2016, **22**, 16089-16098.
26. *CrystalClear-SM Expert v2.1.*, Rigaku Americas, The Woodlands, Texas, USA, and Rigaku Corporation, Tokyo, Japan, 2015.
27. *CrysAlisPro v1.171.41.93a and v1.171.43.109a*, Rigaku Oxford Diffraction, Rigaku Corporation, Tokyo, Japan, 2020-2023.
28. G. M. Sheldrick, SHELXT–Integrated space-group and crystal-structure determination, *Acta Crystallogr., Sect. A: Found. Adv.*, 2015, **71**, 3-8.
29. G. M. Sheldrick, Crystal structure refinement with SHELXL, *Acta Crystallogr., Sect. C: Struct. Chem.*, 2015, **71**, 3-8.
30. A. L. Spek, PLATON SQUEEZE: a tool for the calculation of the disordered solvent contribution to the calculated structure factors, *Acta Crystallogr., Sect. C: Struct. Chem.*, 2015, **71**, 9-18.
31. A. L. Spek, Structure validation in chemical crystallography, *Acta Crystallogr. Sect D: Biol. Crystallogr.*, 2009, **65**, 148-155.
32. O. V. Dolomanov, L. J. Bourhis, R. J. Gildea, J. A. Howard and H. Puschmann, OLEX2: a complete structure solution, refinement and analysis program, *J. Appl. Crystallogr.*, 2009, **42**, 339-341.
33. Y. Xu, Q. Wang, X. Cai, C. Li and Y. Wang, Highly Efficient Electroluminescence from Narrowband Green Circularly Polarized Multiple Resonance Thermally Activated Delayed Fluorescence Enantiomers, *Adv. Mater.*, 2021, **33**, 2100652.
34. Y. Zhang, D. Zhang, T. Huang, A. J. Gillett, Y. Liu, D. Hu, L. Cui, Z. Bin, G. Li, J. Wei and L. Duan, Multi-Resonance Deep-Red Emitters with Shallow Potential-Energy Surfaces to Surpass Energy-Gap Law, *Angew. Chem. Int. Ed.*, 2021, **60**, 20498-20503.
35. J. K. Li, X. Y. Chen, Y. L. Guo, X. C. Wang, A. C. Sue, X. Y. Cao and X. Y. Wang,

B,N-Embedded Double Hetero[7]helicenes with Strong Chiroptical Responses in the Visible Light Region, *J. Am. Chem. Soc.*, 2021, **143**, 17958-17963.

36. S. Y. Yang, S. N. Zou, F. C. Kong, X. J. Liao, Y. K. Qu, Z. Q. Feng, Y. X. Zheng, Z. Q. Jiang and L. S. Liao, A narrowband blue circularly polarized thermally activated delayed fluorescence emitter with a hetero-helicene structure, *Chem. Commun.*, 2021, **57**, 11041-11044.
37. S.-Y. Yang, Q.-S. Tian, X.-J. Liao, Z.-G. Wu, W.-S. Shen, Y.-J. Yu, Z.-Q. Feng, Y.-X. Zheng, Z.-Q. Jiang and L.-S. Liao, Efficient circularly polarized thermally activated delayed fluorescence hetero-[4]helicene with carbonyl-/sulfone-bridged triarylamine structures, *J. Mater. Chem. C*, 2022, **10**, 4393-4401.
38. J. M. dos Santos, D. Sun, J. M. Moreno-Naranjo, D. Hall, F. Zinna, S. T. J. Ryan, W. Shi, T. Matulaitis, D. B. Cordes, A. M. Z. Slawin, D. Beljonne, S. L. Warriner, Y. Olivier, M. J. Fuchter and E. Zysman-Colman, An S-Shaped Double Helicene Showing both Multi-Resonance Thermally Activated Delayed Fluorescence and Circularly Polarized Luminescence, *J. Mater. Chem. C*, 2022, **10**, 4861-4870.
39. W. Yang, N. Li, J. Miao, L. Zhan, S. Gong, Z. Huang and C. Yang, Simple Double Hetero[5]helicenes Realize Highly Efficient and Narrowband Circularly Polarized Organic Light-Emitting Diodes, *CCS Chemistry*, 2022, **4**, 3463-3471.
40. Z. P. Yan, L. Yuan, Y. Zhang, M. X. Mao, X. J. Liao, H. X. Ni, Z. H. Wang, Z. An, Y. X. Zheng and J. L. Zuo, A Chiral Dual-Core Organoboron Structure Realizes Dual-Channel Enhanced Ultrapure Blue Emission and Highly Efficient Circularly Polarized Electroluminescence, *Adv. Mater.*, 2022, **34**, 2204253.
41. Y. Yang, N. Li, J. Miao, X. Cao, A. Ying, K. Pan, X. Lv, F. Ni, Z. Huang, S. Gong and C. Yang, Chiral Multi-Resonance TADF Emitters Exhibiting Narrowband Circularly Polarized Electroluminescence with an EQE of 37.2, *Angew. Chem. Int. Ed.*, 2022, **61**, e202202227.
42. X. Wu, J. W. Huang, B. K. Su, S. Wang, L. Yuan, W. Q. Zheng, H. Zhang, Y. X. Zheng, W. Zhu and P. T. Chou, Fabrication of Circularly Polarized MR-TADF Emitters with Asymmetrical Peripheral-Lock Enhancing Helical B/N-Doped Nanographenes, *Adv.*

Mater., 2022, **34**, 2105080.

43. X. F. Luo, S. Q. Song, X. Wu, C. F. Yip, S. Cai and Y. X. Zheng, A chiral spirofluorene‐embedded multiple‐resonance thermally activated delayed fluorescence emitter for efficient pure‐green circularly polarized electroluminescence, *Aggregate*, 2023, **5**, e445.

44. X. J. Liao, D. Pu, L. Yuan, J. Tong, S. Xing, Z. L. Tu, J. L. Zuo, W. H. Zheng and Y. X. Zheng, Planar Chiral Multiple Resonance Thermally Activated Delayed Fluorescence Materials for Efficient Circularly Polarized Electroluminescence, *Angew. Chem. Int. Ed.*, 2023, **62**, e202217045.

45. Q. Wang, L. Yuan, C. Qu, T. Huang, X. Song, Y. Xu, Y. X. Zheng and Y. Wang, Constructing Highly Efficient Circularly Polarized Multiple-Resonance Thermally Activated Delayed Fluorescence Materials with Intrinsically Helical Chirality, *Adv. Mater.*, 2023, **35**, 2305125.

46. F. Zhang, F. Rauch, A. Swain, T. B. Marder and P. Ravat, Efficient Narrowband Circularly Polarized Light Emitters Based on 1,4-B,N-embedded Rigid Donor-Acceptor Helicenes, *Angew. Chem. Int. Ed.*, 2023, **62**, e202218965.

47. G. Meng, J. Zhou, X. S. Han, W. Zhao, Y. Zhang, M. Li, C. F. Chen, D. Zhang and L. Duan, B-N Covalent Bond Embedded Double Hetero-[n]helicenes for Pure Red Narrowband Circularly Polarized Electroluminescence with High Efficiency and Stability, *Adv. Mater.*, 2024, **36**, 2307420.

48. Z. Ye, H. Wu, Y. Xu, T. Hua, G. Chen, Z. Chen, X. Yin, M. Huang, K. Xu, X. Song, Z. Huang, X. Lv, J. Miao, X. Cao and C. Yang, Deep-Blue Narrowband Hetero[6]helicenes Showing Circularly Polarized Thermally Activated Delayed Fluorescence Toward High-Performance OLEDs, *Adv. Mater.*, 2024, **36**, 2308314.

49. G. Venugopal, V. Kumar, A. Badrinarayan Jadhav, S. D. Dongre, A. Khan, R. Gonnade, J. Kumar and S. Santhosh Babu, Boron- and Oxygen-Doped pi-Extended Helical Nanographene with Circularly Polarised Thermally Activated Delayed Fluorescence, *Chem. Eur. J.*, 2024, **30**, e202304169.

50. W. C. Guo, W. L. Zhao, K. K. Tan, M. Li and C. F. Chen, B,N-Embedded

Hetero[9]helicene Toward Highly Efficient Circularly Polarized Electroluminescence, *Angew. Chem. Int. Ed.*, 2024, **63**, e202401835.

51. Y. Xu, H. Hafeez, J. Seibert, S. Wu, J. S. O. Ortiz, J. Crassous, S. Bräse, I. D. W. Samuel and E. Zysman‐Colman, [2.2]Paracyclophane‐Substituted Chiral Multiresonant Thermally Activated Delayed Fluorescence Emitters for Efficient Organic Light‐Emitting Diodes, *Adv. Funct. Mater.*, 2024, DOI: 10.1002/adfm.202402036, 2402036.

52. L. Yuan, Y. F. Yang, Z. P. Yan, J. J. Hu, D. Mao, H. X. Ni and Y. X. Zheng, Circularly Polarized Electroluminescence from Intrinsically Axial Chiral Materials Based on Bidibenzo[b,d]furan/bidibenzo[b,d]thiophene, *Adv. Funct. Mater.*, 2024, 2403803.

53. L. Yuan, J. W. Xu, Z. P. Yan, Y. F. Yang, D. Mao, J. J. Hu, H. X. Ni, C. H. Li, J. L. Zuo and Y. X. Zheng, Tetraborated Intrinsically Axial Chiral Multi-resonance Thermally Activated Delayed Fluorescence Materials, *Angew. Chem. Int. Ed.*, 2024, **63**, e202407277.

54. S. Xing, X. S. Zhong, X. J. Liao, Y. Wang, L. Yuan, H. X. Ni and Y. X. Zheng, Axially Chiral Multiple Resonance Thermally Activated Delayed Fluorescence Enantiomers for Efficient Circularly Polarized Electroluminescence, *Adv. Optical Mater.*, 2024, **12**, 2400685.

55. S.-Q. Song, X. Han, Z.-Z. Huo, C.-F. Yip, X.-F. Hong, M.-N. Ding and Y.-X. Zheng, Chiral multiple-resonance thermally activated delayed fluorescence materials based on chiral spiro-axis skeleton for efficient circularly polarized electroluminescence, *Sci. China Chem.*, 2024, **67**, 2257-2264.

56. Y. Wang, Z. Y. Lv, Z. X. Chen, S. Xing, Z. Z. Huo, X. F. Hong, L. Yuan, W. Li and Y. X. Zheng, Multiple-resonance thermally activated delayed fluorescence materials based on phosphorus central chirality for efficient circularly polarized electroluminescence, *Mater. Horiz.*, 2024, DOI: 10.1039/d4mh00605d.
